# Supplementary material for: Ancient diversity in host-parasite interaction genes in a model parasitic nematode
Source: Nat Commun. 2023 Nov 27;14:7776. doi: 10.1038/s41467-023-43556-w (PMC10682056; doi:10.1038/s41467-023-43556-w)
Supplement: Supplementary file 1 — Supplementary Information [file 41467_2023_43556_MOESM1_ESM.pdf]

# Supplementary information for *Ancient diversity in host-parasite interaction genes in a model parasitic nematode*

Lewis Stevens[1], Isaac Martinez-Ugalde[2], Erna King[1], Martin Wagah[1], Dominic Absolon[1], Rowan Bancroft[2], Pablo Gonzalez de la Rosa[1], Jessica L Hall[2], Manuela Kieninger[1], Agnieszka Kloch[3], Sarah Pelan[1], Elaine Robertson[4], Amy B Pedersen[2], Cei Abreu-Goodger[2], Amy H Buck[4], Mark Blaxter[1]

1. Tree of Life, Wellcome Sanger Institute, Hinxton, UK

2. Institute of Ecology and Evolution, School of Biological Sciences, University of Edinburgh, Edinburgh, UK

3. Faculty of Biology, University of Warsaw, Warsaw, Poland

4. Institute of Immunology & Infection Research, School of Biological Sciences, University of Edinburgh, Edinburgh, UK

**Supplementary Figure 1: Blob plot of Heligmosomoides polygyrus ngHelPoly2 dataset 3**
**Supplementary Figure 2: K-mer profiles of PacBio HiFi data for all Heligmosomoides bakeri and Heligmosomoides polygyrus individuals 4**
**Supplementary Figure 3: Chromosome-level reference genomes for Heligmosomoides bakeri and Heligmosomoides polygyrus 5**
**Supplementary Figure 4: Telomeric repeat sequence in Heligmosomoides reference genomes 6**
**Supplementary Figure 5: GC-coverage bias in the H. bakeri nxHelBake1 and H. polygyrus ngHelPoly1 PiMmS data 7**
**Supplementary Figure 6: Cytochrome oxidase 1 (COI) phylogeny of Heligmosomoides and related nematodes 8**
**Supplementary Figure 7: Repeat content and synteny in the H. bakeri and H. polygyrus X chromosomes 9**
**Supplementary Figure 8: Synonymous site divergence between five nematode sister species pairs 10**
**Supplementary Figure 9: Distribution of heterozygous SNPs in Heligmosomoides bakeri and Heligmosomoides polygyrus 11**
**Supplementary Figure 10: Example hyper-divergent haplotype on H. bakeri nxHelBake1 chromosome V 12**
**Supplementary Figure 11: Optimising the hyper-divergent region calling pipeline 13**
**Supplementary Figure 12: Locations of hyper-divergent haplotypes across all three individuals 14**
**Supplementary Figure 13: Gene ontology (GO) enrichment for hyper-divergent haplotypes 16**
**Supplementary Figure 14: Read alignments in a region in nxHelBake1.1 containing two Ancylostoma-secreted protein homologues showing evidence of trans-specific polymorphism 17**
**Supplementary Figure 15: RNA-seq support for genes predicted in the alternate H. bakeri haplotype 18**
**Supplementary Figure 16: Read alignments in a region in ngHelPoly1.1 containing two Ancylostoma-secreted proteins showing evidence of trans-specific polymorphism 19**
**Supplementary Figure 17: Trans-specific polymorphism in novel secreted proteins homologs 20**
**Supplementary Figure 18: Trans-specific polymorphism in a region containing multiple transthyretin-like proteins 21**
**Supplementary Figure 19: Trans-specific polymorphism in a homolog of H11 aminopeptidase 22**
**Supplementary Figure 20: Shared haplotypes show similar levels of divergence to the genome-wide average 23**
**Supplementary Table 1: Single-worm sequencing and assembly metrics 24**
**Supplementary Table 2: Protein-coding gene prediction metrics 25**
**Supplementary Table 3: Heterozygosity in the H. bakeri and H. polygyrus genomes 26**
**Supplementary Table 4: H. bakeri hyper-divergent haplotype metrics 27**
**Supplementary Table 5: GO terms significantly enriched in hyper-divergent haplotypes 28**
**Supplementary Table 6: Accessions of the data used in gene prediction and phylogenomic analyses 29**
**Supplementary Table 7: Heligmosomum genome assembly metrics 30**
**Supplementary Table 8: Genome assembly accession numbers 31**

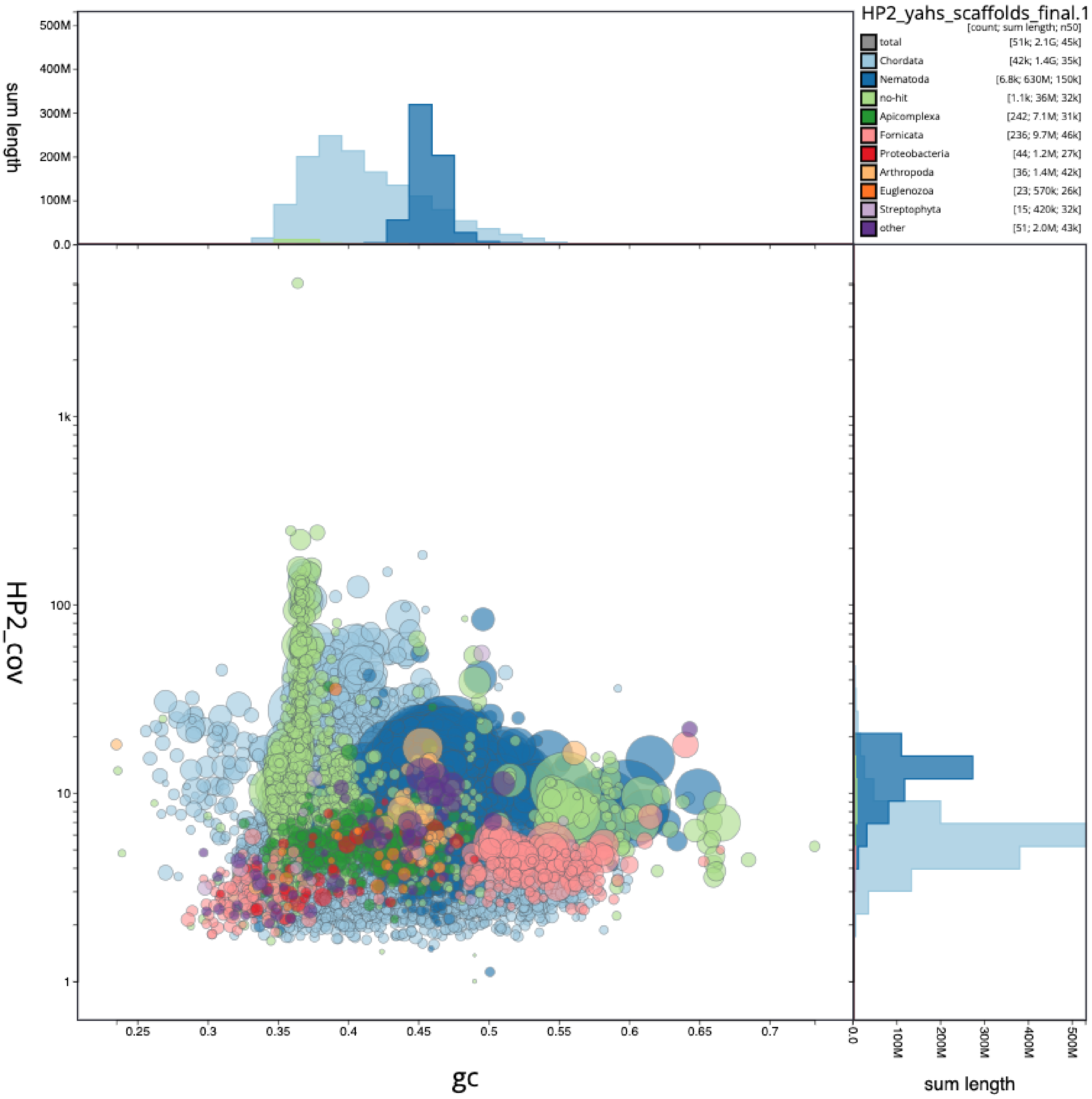


**Supplementary Figure 1: Blob plot of *Heligmosomoides polygyrus* ngHelPoly2 dataset**

Blob plot of the assembly of the *H. polygyrus* ngHelPoly2 dataset showing extensive contamination by host and two diplomonad parasites (a *Giardia* sp. and a *Spironucleus* sp.). Scaffolds are plotted based on their GC content (x-axis) and their PacBio HiFi read coverage (y-axis). Scaffolds are coloured by phylum of their best BLAST match in the NR NCBI database. Circles are sized in proportion to scaffold length on a square-root scale. Histograms show the distribution of scaffold length sum along each axis. Two groups of scaffolds are likely derived from Fornicata (Metamonada) parasites: the leftmost blob corresponds to a *Spironucleus* sp. and the rightmost blob corresponds to a *Giardia* sp. Note that scaffolds labelled as "Apicomplexa" are mislabelled *Apodemus sylvaticus* host scaffolds; all hits are to a *Plasmodium yoelii yoelii* genome in NCBI (GCA_000003085.2) which is contaminated with host rodent sequence.

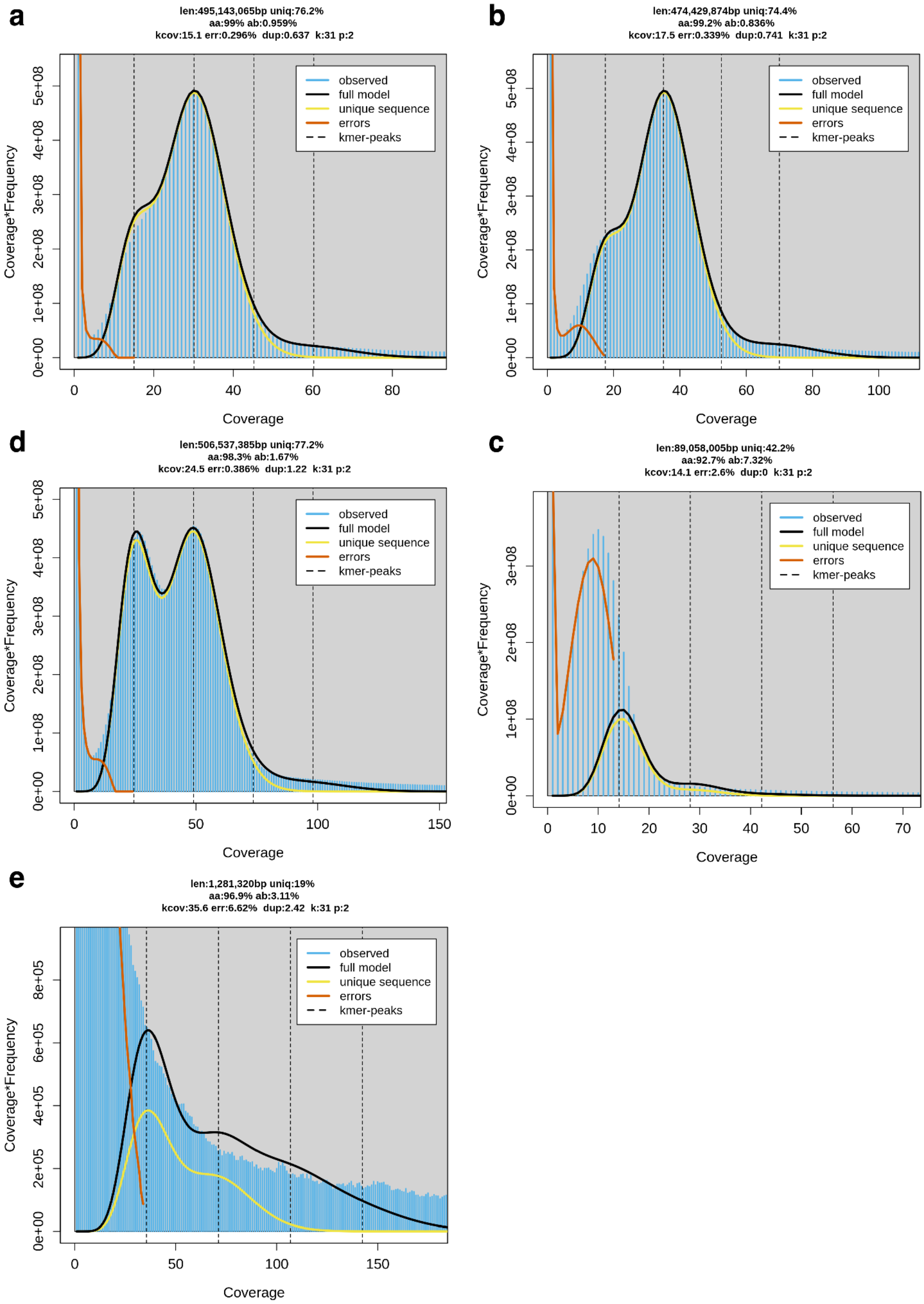


**Supplementary Figure 2: *K*-mer profiles of PacBio HiFi data for all *Heligmosomoides bakeri* and *Heligmosomoides polygyrus* individuals**

*K*-mer profiles (k = 31) and fitted models for PacBio raw HiFi read data from GenomeScope 2.0 for (a) *H. bakeri* nxHelBake1, (b) *H. bakeri* nxHelBake2, (c) *H. bakeri* nxHelBake3, (d) *H. polygyrus* ngHelPoly1 and (e) *H. polygyrus* ngHelPoly2. The *k*-mer profile shown for *H. polygyrus* ngHelPoly2 is of the read set after removing reads that map to *Apodemus sylvaticus* and *Giardia muris*. The genome size and heterozygosity estimates for *H. bakeri* nxHelBake3 (c) and *H. polygyrus* ngHelPoly2 (e) are not reliable due to low coverage. In the high coverage datasets, we note that genome size estimates are substantially less than the assembled reference genomes, which may be caused by high copy number *k*-mers being poorly modelled.

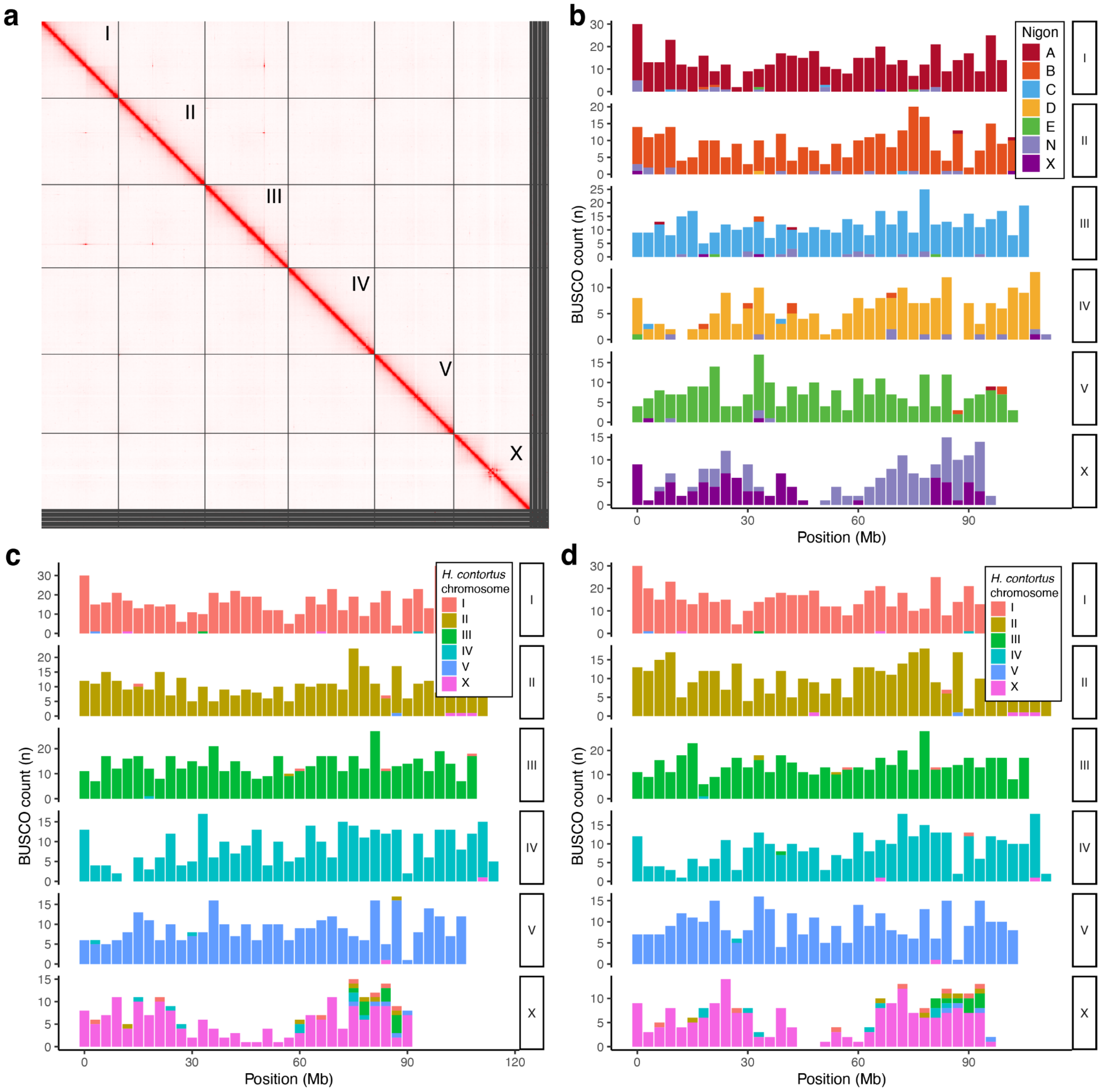


**Supplementary Figure 3: Chromosome-level reference genomes for *Heligmosomoides bakeri* and *Heligmosomoides polygyrus***

(a) Hi-C contact map for ngHelPoly1.1 reference genome. Chromosome names are indicated. (b) Distribution of BUSCO genes in the six ngHelPoly1.1 chromosomes coloured by Nigon element (Gonzalez de la Rosa *et al.* 2021). Distribution of BUSCO genes coloured by their location in the chromosome-level *H. contortus* (PRJEB506) genome in the (c) *H. bakeri* nxHelBake1.1 and (c) *H. polygyrus* ngHelPoly1.1 reference genomes. Source data for figures b-d can be found in the Source Data file.

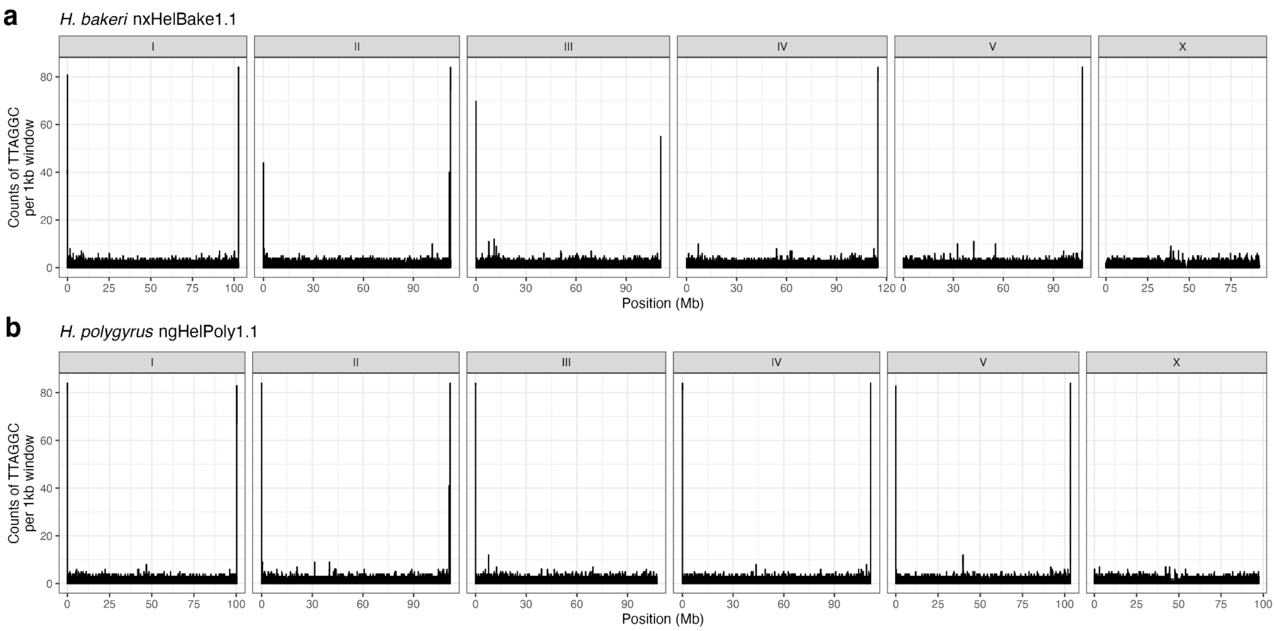


**Supplementary Figure 4: Telomeric repeat sequence in *Heligmosomoides* reference genomes**

Counts of the nematode telomeric repeat sequence (TTAGGC) in 1 kb windows in the (a) *H. bakeri* nxHelBake1 and (b) *H. polygyrus* ngHelPoly1 reference genomes. Telomeric repeat counts are shown for the six chromosome-sized scaffolds only. Source data for this figure can be found in the GitHub repository.

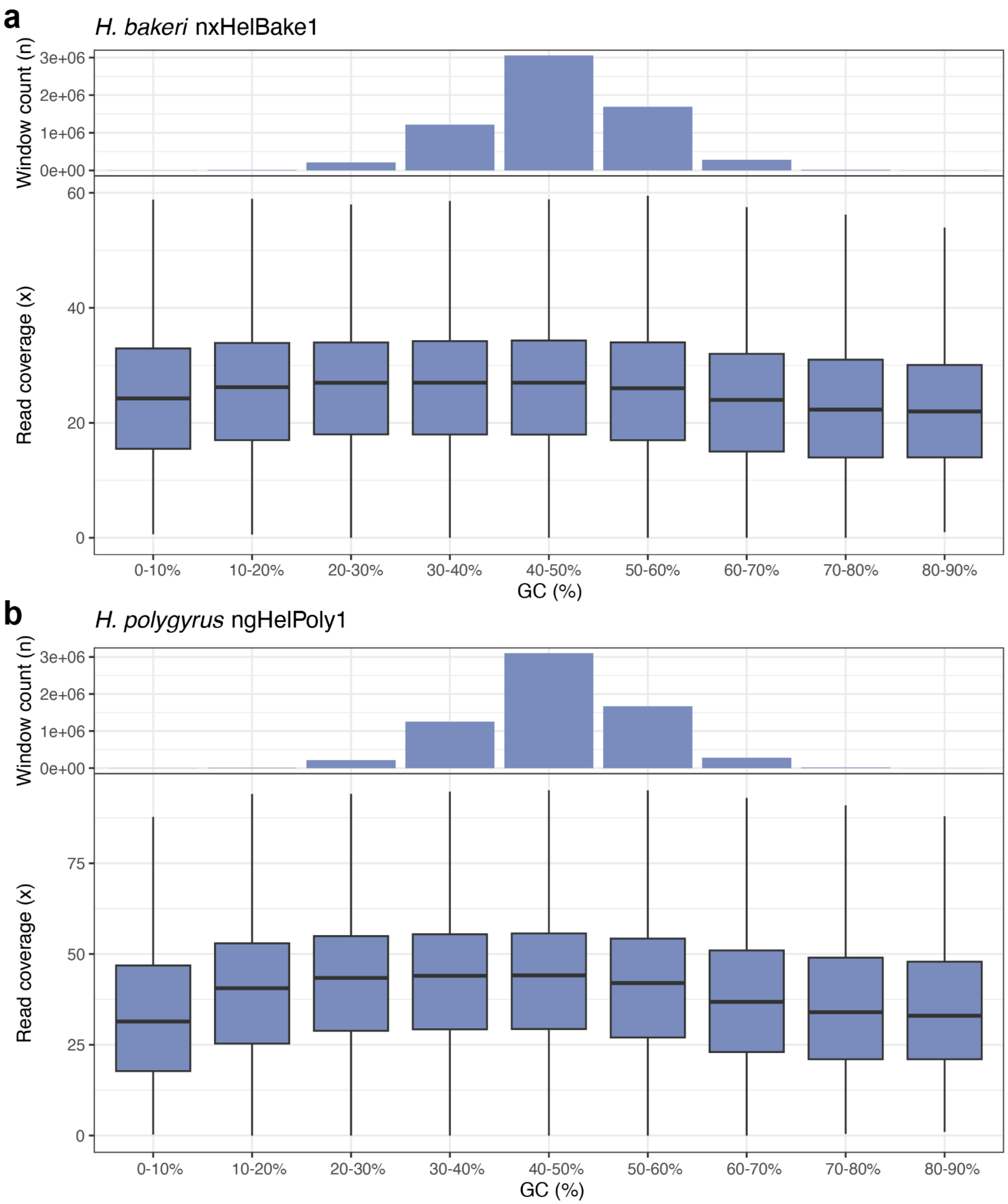


**Supplementary Figure 5: GC-coverage bias in the *H. bakeri* nxHelBake1 and *H. polygyrus* ngHelPoly1 PiMmS data**

PacBio HiFi read coverage in 100 bp windows across the (a) *H. bakeri* nxHelBake1 and (b) *H. polygyrus* ngHelPoly1 reference genomes, binned by GC %. Histograms show the counts of windows in each bin. Only windows that were 100 bp in length and that had ≤ 50% Ns are shown. No windows in either genome had a GC% of > 90%. Source data for this figure can be found in the GitHub repository.

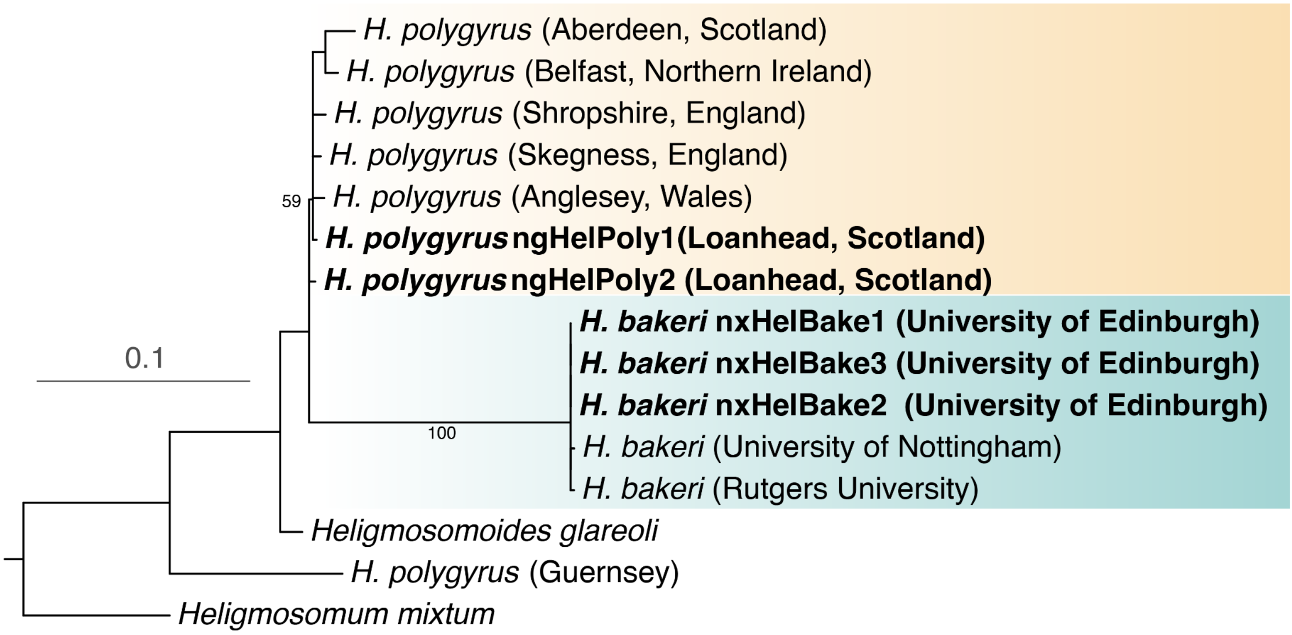


**Supplementary Figure 6: Cytochrome oxidase 1 (COI) phylogeny of Heligmosomoides and related nematodes**

Maximum likelihood phylogeny of the mitochondrial cytochrome oxidase 1 gene in laboratory isolates of *H. bakeri*, wild isolates of *H. polygyrus*, and outgroup taxa. The sequences are derived from *Cable et al.* (2006) or the mitochondrial genomes of individuals sequenced as part of this work (highlighted in bold). The origin of each isolate is shown in parentheses. Bootstrap support values are shown for the branches subtending the *H. bakeri* and *H. polygyrus* clades. Branch lengths represent the number of substitutions per site; scale is shown. As noted by Cable *et al.* (2006) and Maizels *et al.* (2011), the COI sequence from the "*H. polygyrus*" isolate from Guernsey is highly divergent from other *H. polygyrus* isolates. We believe this is caused either by misidentification or a low-quality sequence. Source data for this figure can be found in the GitHub repository.

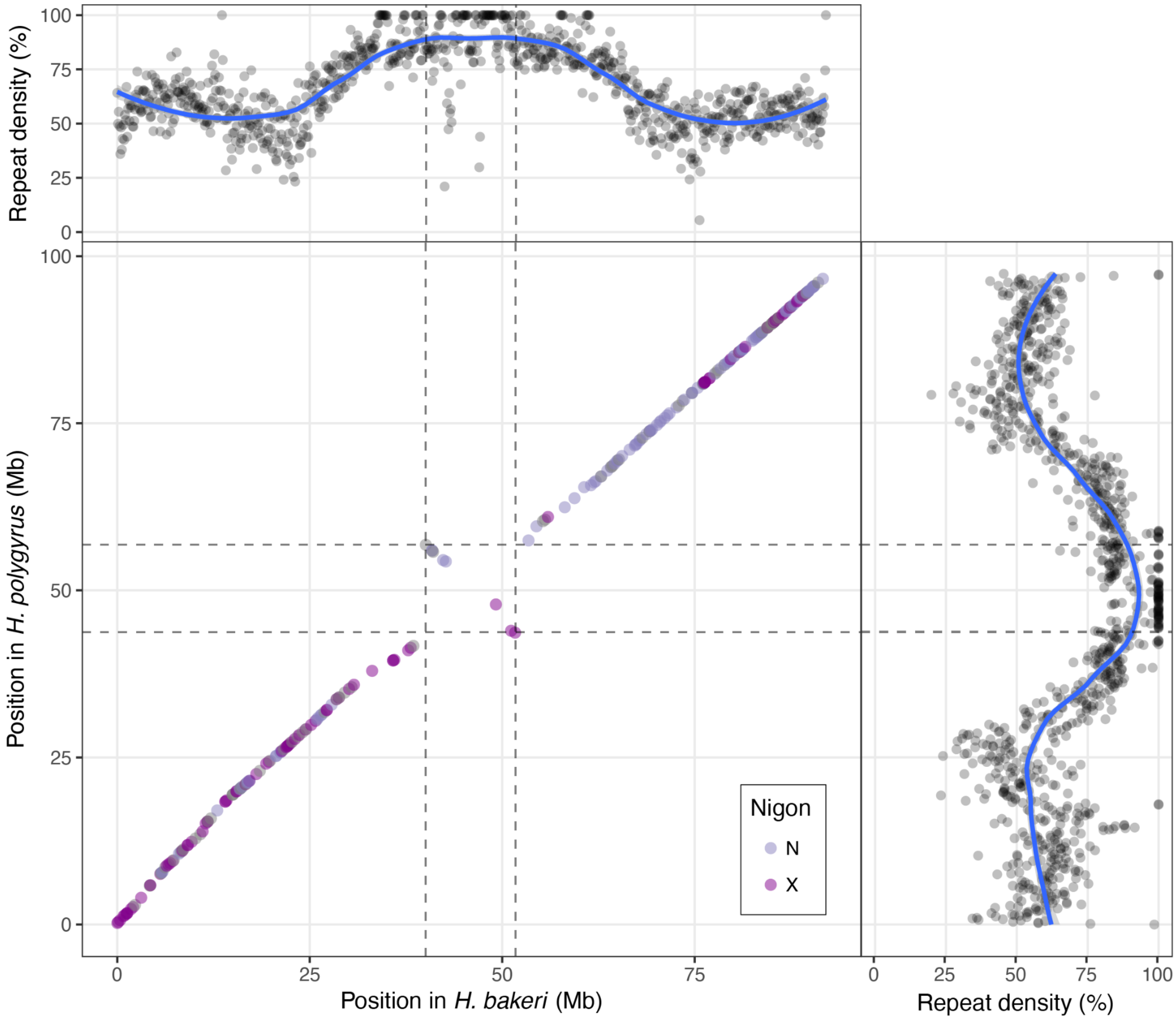


**Supplementary Figure 7: Repeat content and synteny in the *H. bakeri* and *H. polygyrus* X chromosomes**

Lower left panel: The relative position for 260 BUSCO genes in *H. bakeri* and *H. polygyrus* X chromosomes are shown as dots. The dots are coloured by their allocation to Nigon elements. Right panel and upper panel: Repeat content in 100 kb windows is shown for both X chromosomes; lines represent LOESS smoothing functions fitted to the data. The location of an apparent inversion is indicated with dotted lines. The inversion-containing regions in both X chromosomes are highly repetitive in both genomes and the order of the contigs within these regions are uncertain in our reference genomes. Source data for this figure can be found in the GitHub repository.

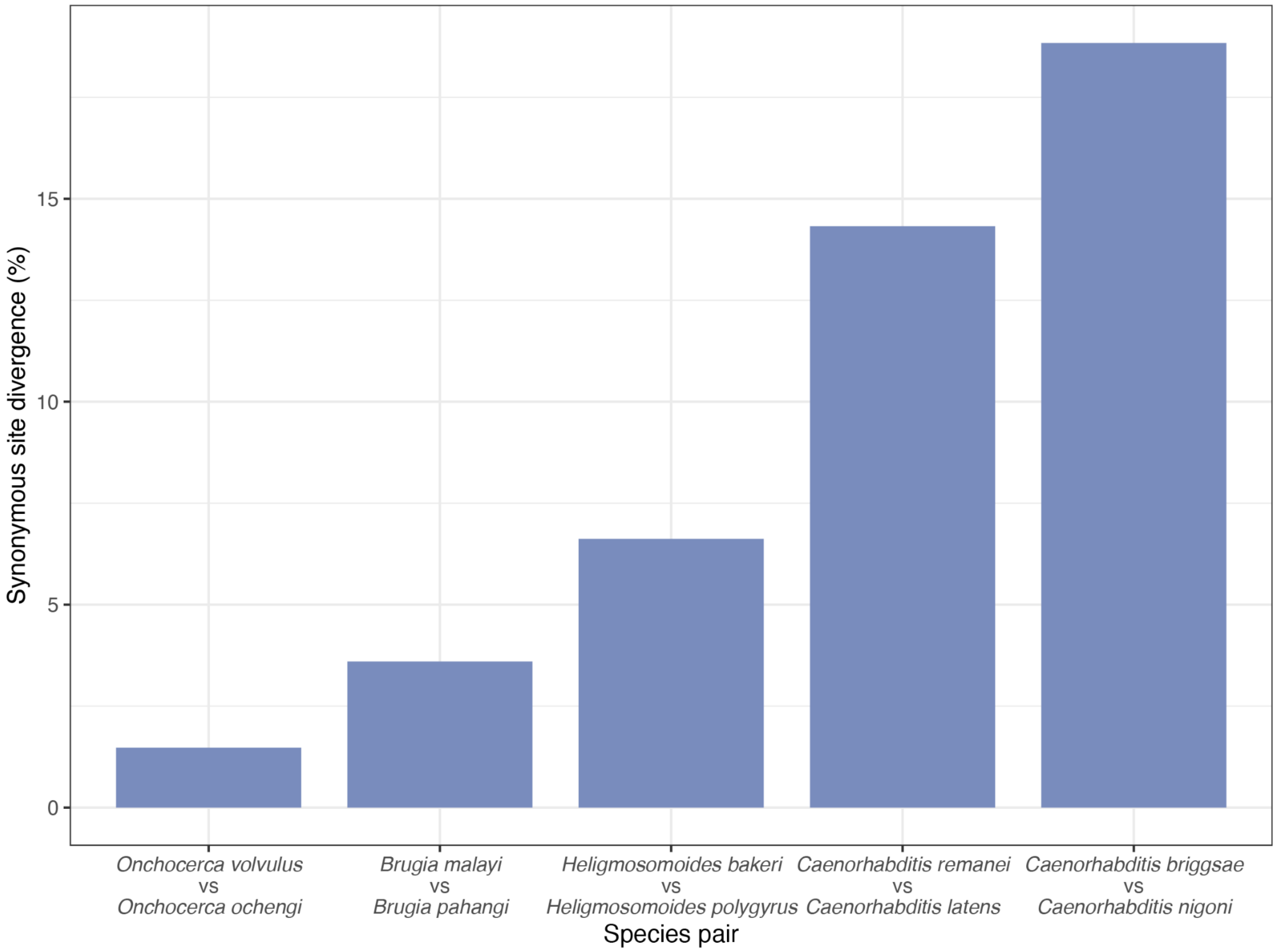


**Supplementary Figure 8: Synonymous site divergence between five nematode sister species pairs**

Mean synonymous site divergence between five nematode sister species. Single-copy orthologues were identified using BUSCO (with the nematoda_odb10 dataset); orthologue counts ranged from 2,539 to 2,954. Synonymous site divergence was calculated for each pair of orthologues using the Nei-Gojobori method implemented in codeml. Orthologues that had a divergence greater than 80% were filtered out before calculating the mean. Source data for this figure can be found in the Source Data file.

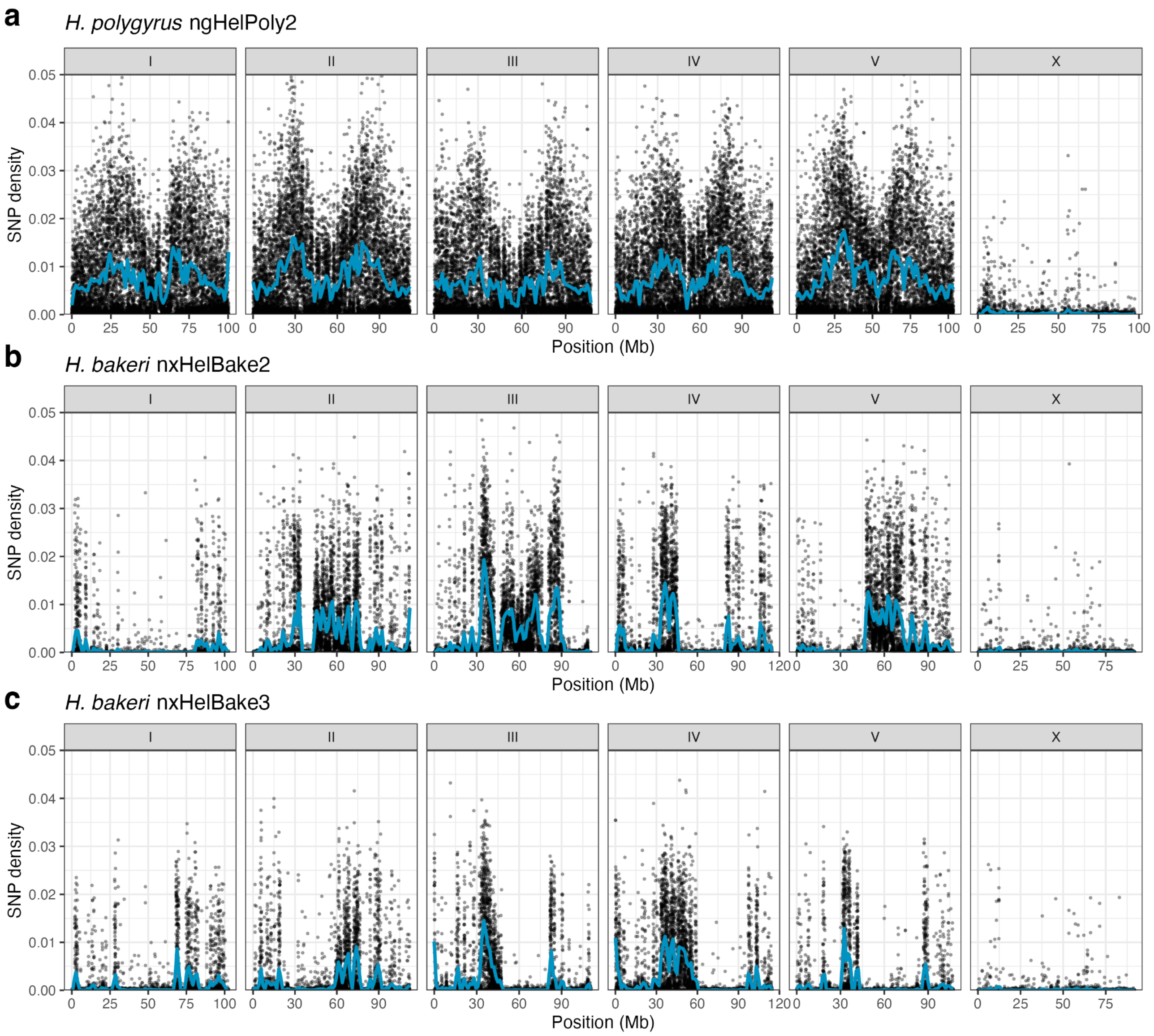


**Supplementary Figure 9: Distribution of heterozygous SNPs in *Heligmosomoides bakeri* and *Heligmosomoides polygyrus***

Distribution of heterozygous SNPs in (a) *H. polygyrus* ngHelPoly2 relative to the ngHelPoly1.1 reference genome and the (b) *H. bakeri* nxHelBake2 and (c) *H. bakeri* nxHelBake3 relative to the nxHelBake1 reference genome. Points represent the density of biallelic SNPs in 10 kb windows. All three individuals were male and therefore the X chromosome is hemizygous; the SNP density peaks on the X chromosome are therefore erroneous and are a consequence of mismapped PacBio HiFi reads. SNPs called in repeat-containing regions were filtered and SNP density was calculated as the number of non-repetitive SNPs per non-repetitive base. Homozygous alternate variants (i.e. variants that represented differences from the reference genome rather than heterozygous SNPs) were ignored. Lines represent LOESS smoothing curves fitted to the data. Source data for this figure can be found in the GitHub repository.

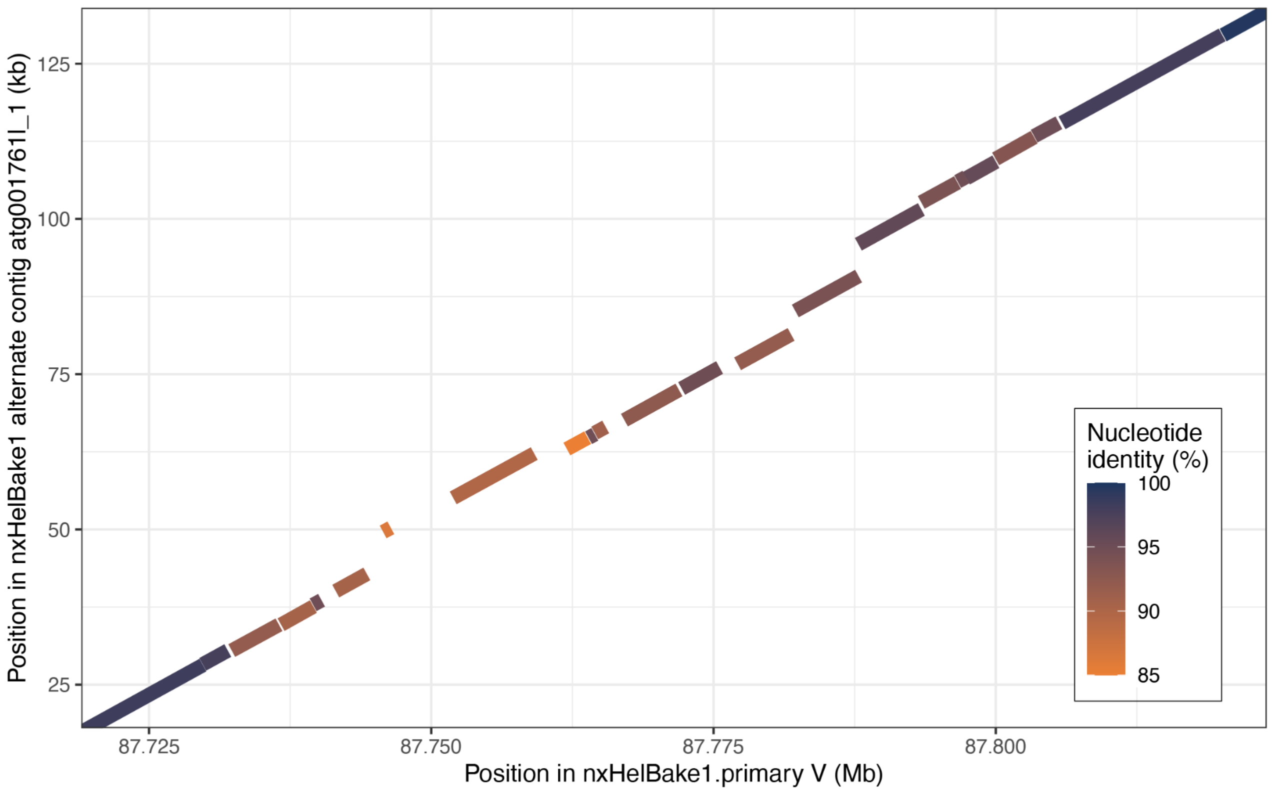


**Supplementary Figure 10: Example hyper-divergent haplotype on *H. bakeri* nxHelBake1 chromosome V**

Nucleotide alignment between nxHelBake1 alternate contig atg001761l_1 and nxHelBake1 primary chromosome V (87.71 - 87.86) with each aligned segment coloured by its nucleotide identity. Repetitive alignments are not shown. The two non-divergent flanking alignments show high nucleotide identity (99.48% and 99.73%, respectively) whereas several aligned segments within the hyper-divergent haplotype have nucleotide identities of < 90%. Read alignments for this region are shown in Figure 3C. Source data for this figure can be found in the GitHub repository.

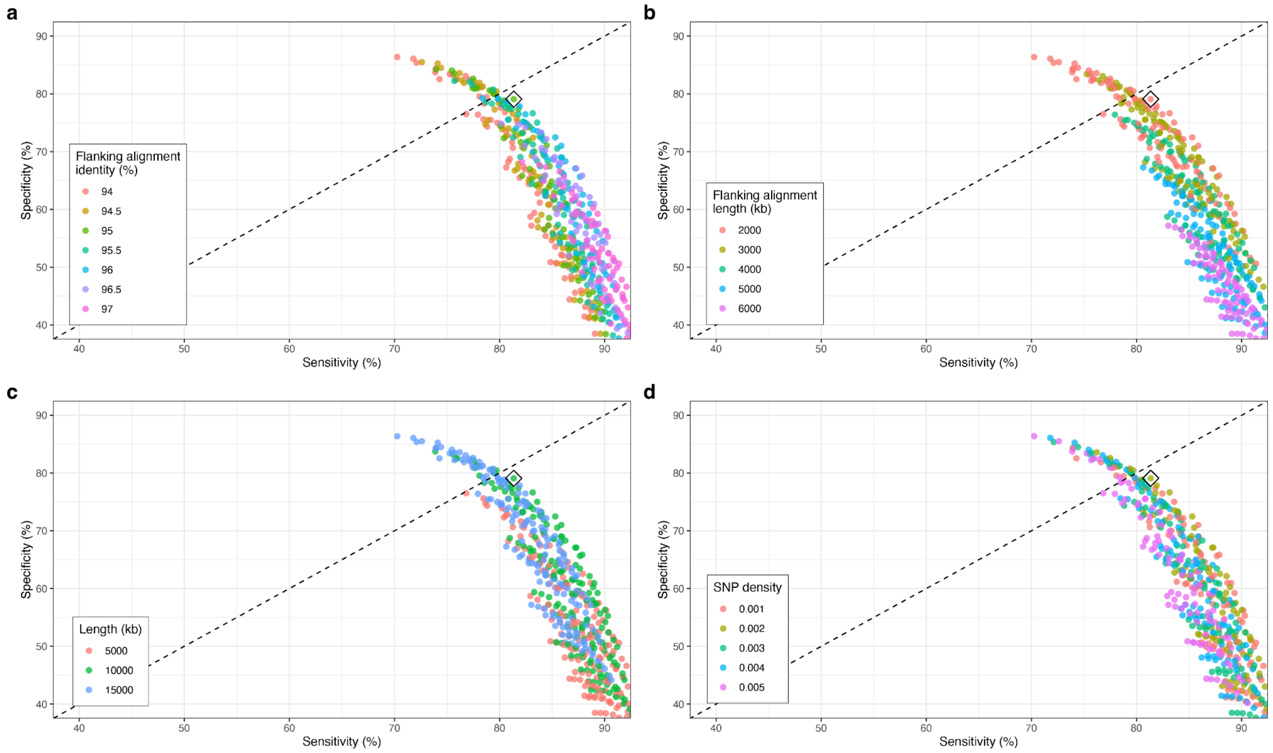


**Supplementary Figure 11: Optimising the hyper-divergent region calling pipeline**
Each point represents an independent run of the hyper-divergent haplotype calling pipeline on PacBio CLR assemblies for 15 *C. elegans* strains (Lee *et al.* 2021) using a different parameter set. The output of each run is summarised by sensitivity on the X-axis (the number of overlapping bases divided by the total number of bases defined by Lee *et al.* (2021) and specificity (the number of overlapping bases divided by the total number of bases classified as hyper-divergent by our approach). Each panel represents different parameter: (a) flanking alignment identities of 94-97%, (b) flanking alignment lengths of 2-6 kb, (c) minimum size of alignment gaps to be considered as a hyper-divergent haplotype of 5-15 kb, and (d) SNP density, derived from assembly-based variant calling, within alignment gaps of 0.001-0.005. The diamond represents the chosen parameter set. Source data for this figure can be found in the Source Data file.

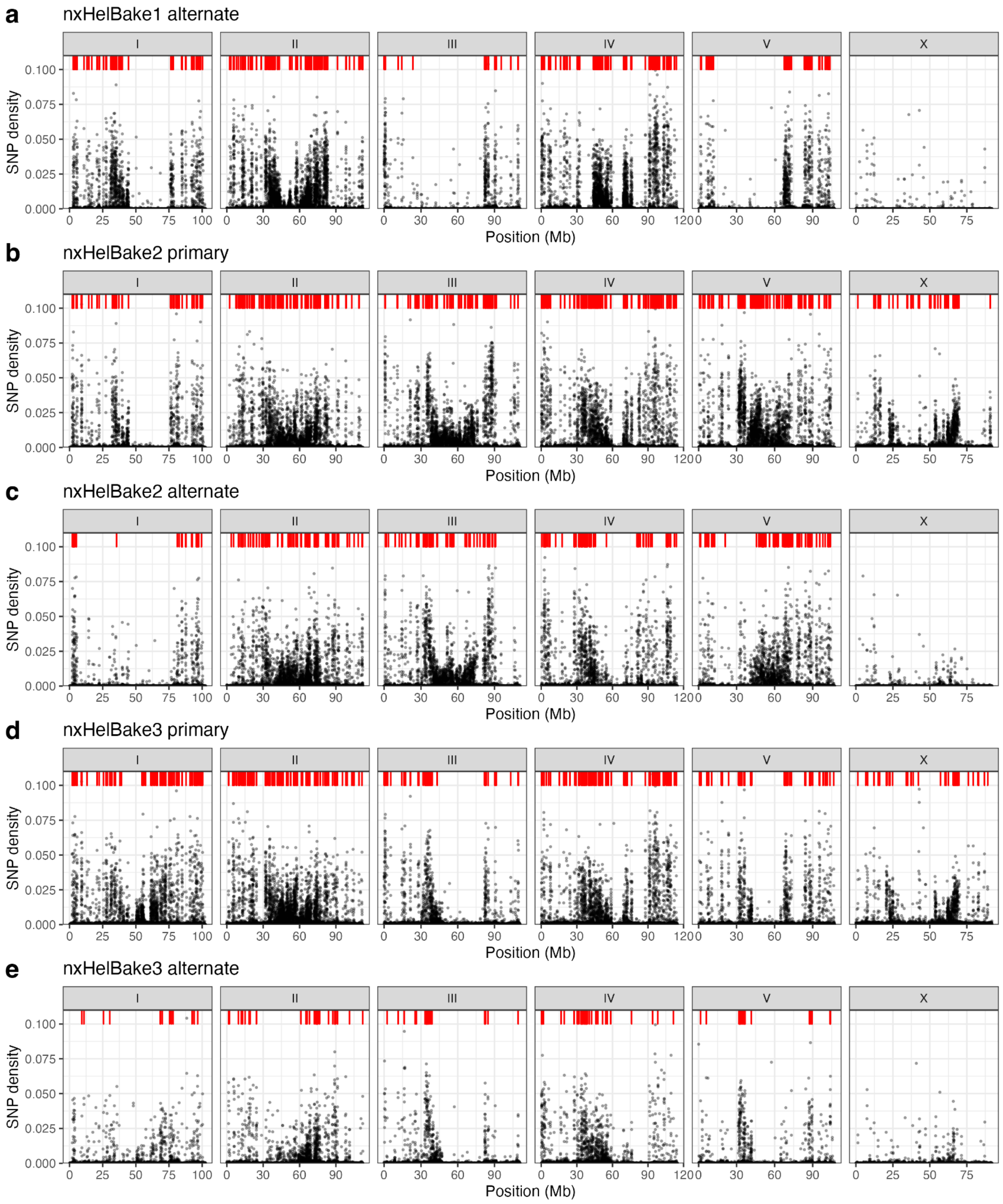


**Supplementary Figure 12: Locations of hyper-divergent haplotypes across all three individuals**

The locations of hyper-divergent haplotypes in all five non-reference haplotypes: (a) nxHelBake1 alternate, (b) nxHelBake2 primary, (c) nxHelBake2 alternate, (d) nxHelBake3 primary, (e) nxHelBake3 alternate. Red boxes represent locations of hyper-divergent haplotypes. Hyper-divergent haplotypes called on the X chromosome in the three alternate haplotypes were removed (2, 1, and 1 from nxHelBake1, nxHelBake2, and nxHelBake3 alternate assemblies, respectively). The distribution of heterozygous SNPs, derived from assembly-based variant calling, in 10 kb windows are shown for each haplotype. SNPs that overlapped with a repeat annotation were removed and SNP density was calculated using the remaining SNPs and the number of

non-repetitive bases in each window. Source data for this figure can be found in the GitHub repository.

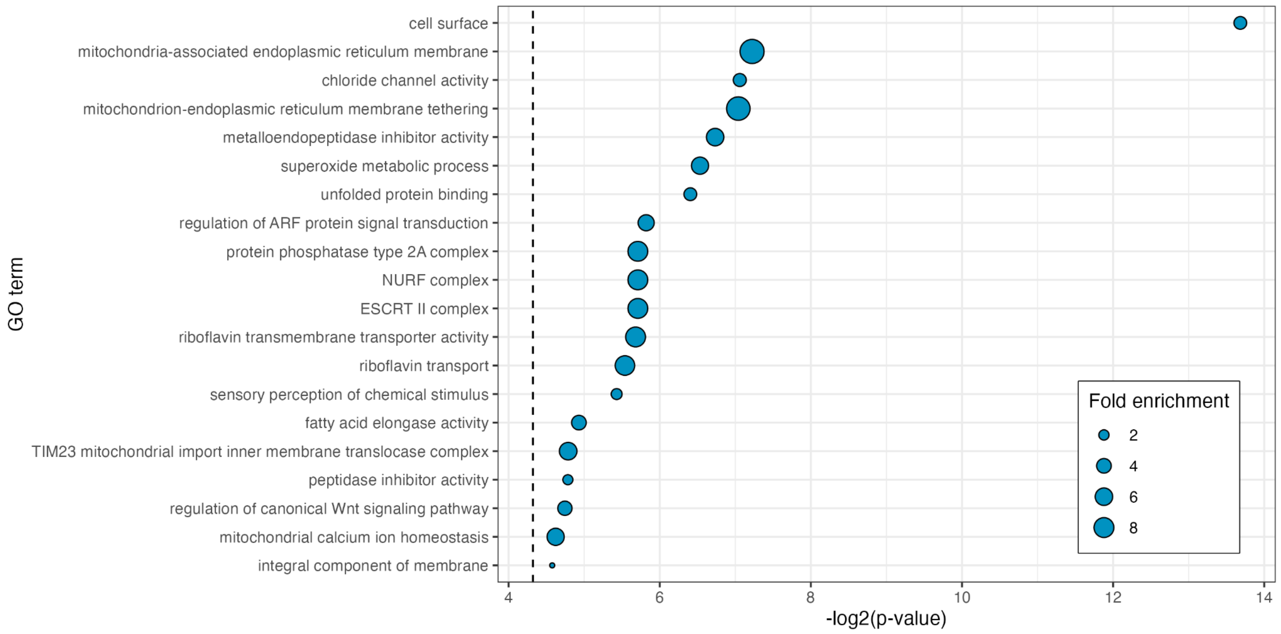


**Supplementary Figure 13: Gene ontology (GO) enrichment for hyper-divergent haplotypes**

Gene ontology (GO) enrichment for the 1,734 genes found in hyper-divergent haplotypes in *H. bakeri*. GO terms from all three ontologies (‘molecular function’, ‘biological process’, and ‘cellular component’) are shown. P-values were calculated using the ‘weight01’ algorithm from TopGO which considers the GO hierarchy. Circles are scaled by the fold enrichment in hyper-divergent haplotypes. The dotted line represents a significance threshold of 0.05. Source data for this figure can be found in Supplementary Table 5.

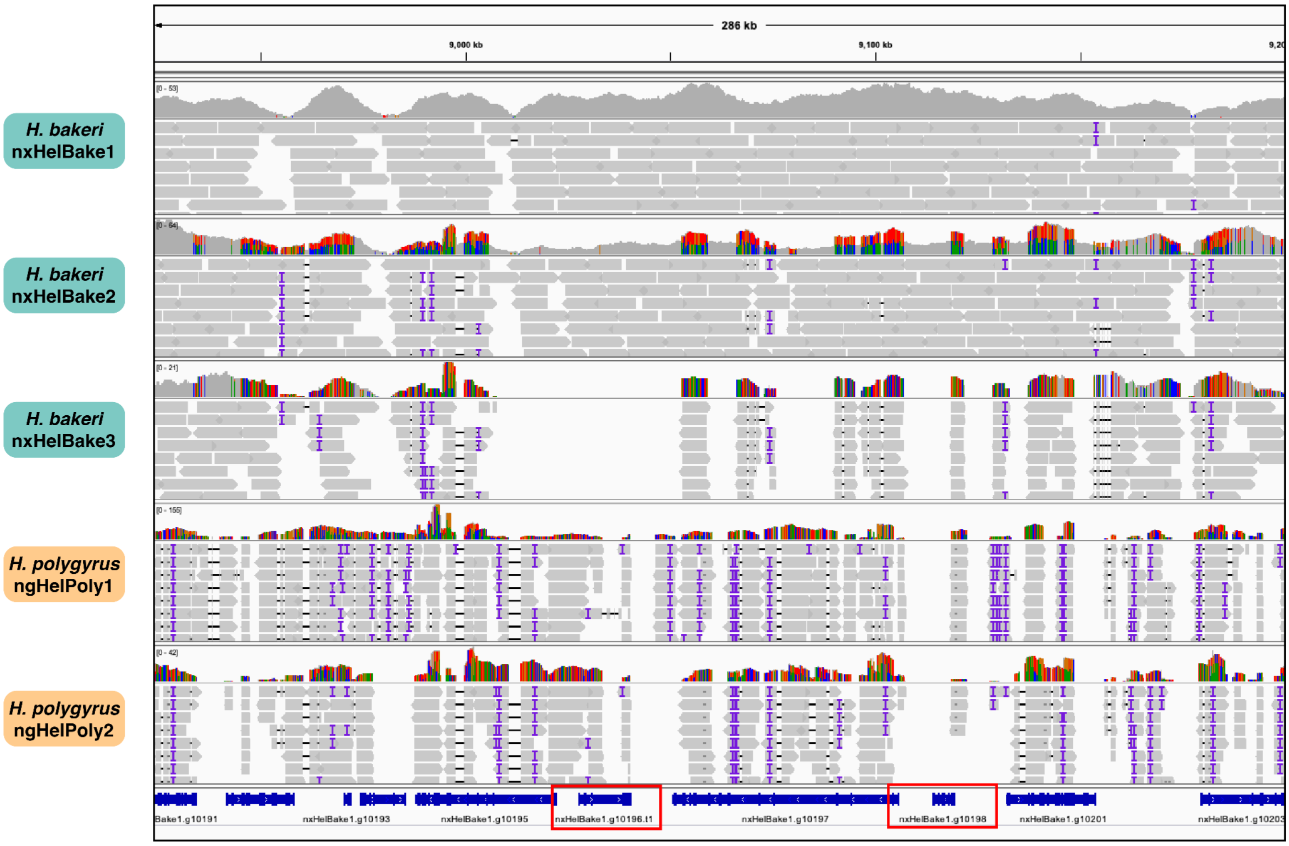


**Supplementary Figure 14: Read alignments in a region in nxHelBake1.1 containing two *Ancylostoma*-secreted protein homologues showing evidence of trans-specific polymorphism**

PacBio HiFi read alignments of all sequenced individuals to the nxHelBake1.1 reference genome in a 287 kb region on chromosome I (I:8.92-9.19 Mb) showing evidence of trans-specific polymorphisms. Two genes in this region belong to the ASP family (nxHelBake1.g10196 and nxHelBake1.g10198; highlighted in red boxes). The top panel shows the coverage and the bottom panel shows aligned PacBio HiFi reads. The coloured vertical lines indicate mismatched bases at that position. Note that mismatched bases are only shown in the coverage tracks and not in the read alignments at this zoom level in IGV.

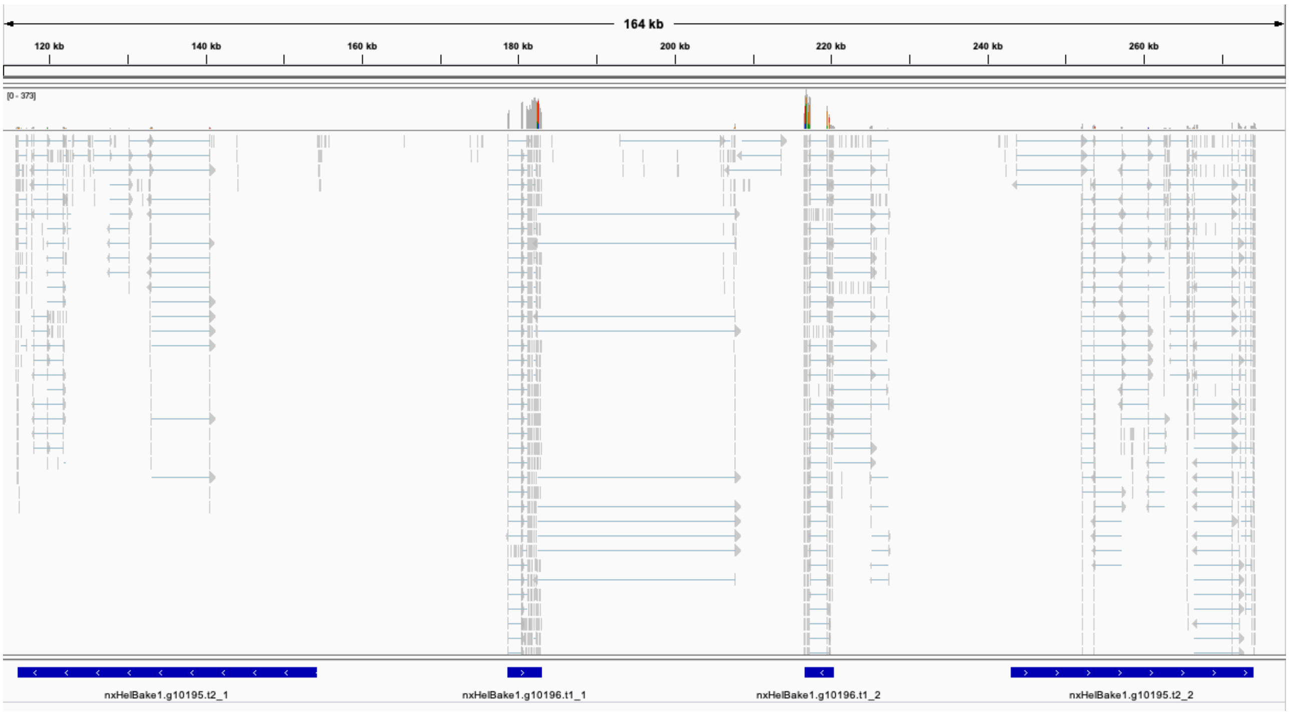


**Supplementary Figure 15: RNA-seq support for genes predicted in the alternate *H. bakeri* haplotype**

Alignments of short-read RNA-seq data collected from pools of *H. bakeri* individuals (Rausch *et al.* 2018) to the alternate haplotype from the nxHelBake2 primary assembly. The top panel shows the coverage and the bottom panel shows aligned RNA-seq reads. Both homologues of nxHelBake1.g10195 (which contains a growth factor receptor domain) and both homologues of the ASP nxHelBake1.g10196 (a member of the *Ancylostoma*-secreted protein family) are supported by RNA-seq reads.

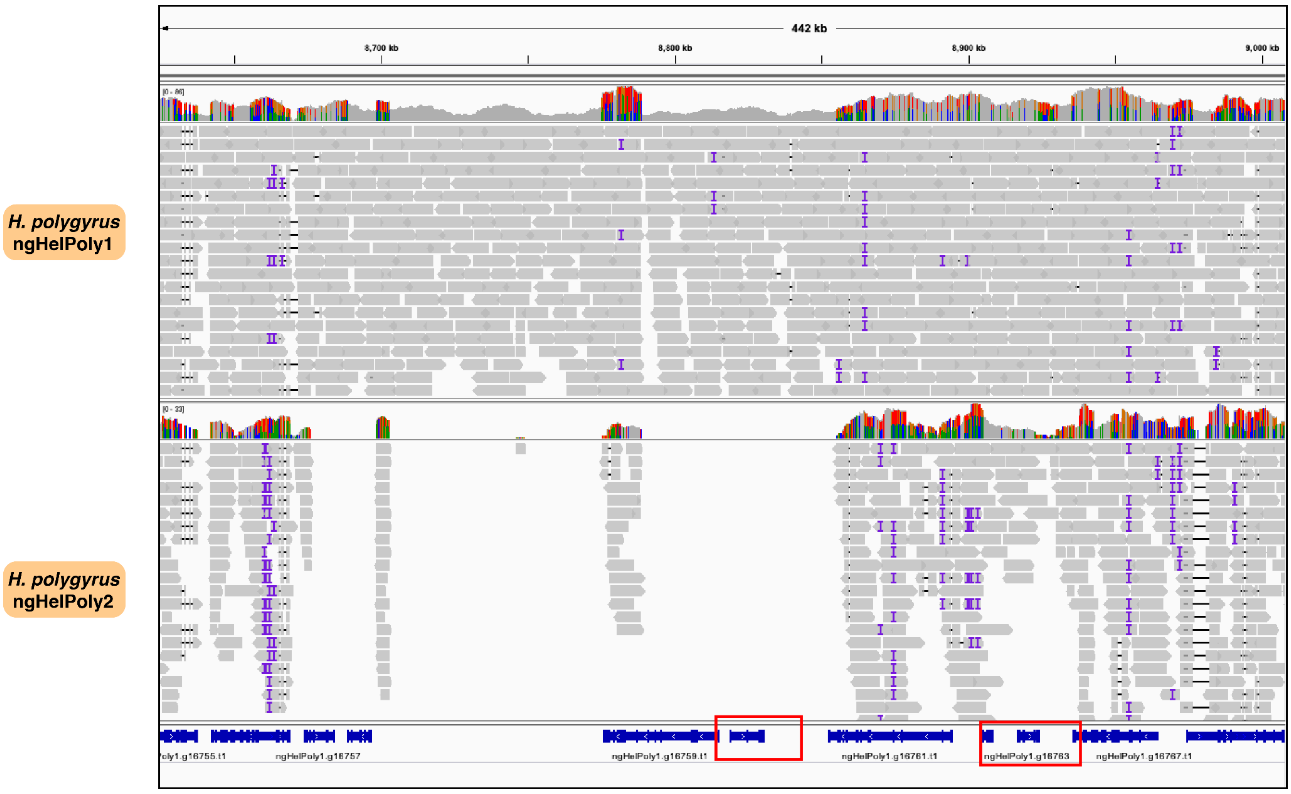


**Supplementary Figure 16: Read alignments in a region in ngHelPoly1.1 containing two *Ancylostoma*-secreted proteins showing evidence of trans-specific polymorphism**

PacBio HiFi read alignments of both *H. polygyrus* individuals to the ngHelPoly1.1 reference genome in a ~383 kb region on chromosome I (I:8.62-9.01 Mb) showing evidence of trans-specific polymorphisms. Two genes in this region belong to the ASP family (ngHelPoly1.g16760.t1 and ngHelPoly1.g16763.t1; highlighted with red boxes). The top panel shows the coverage and the bottom panel shows aligned PacBio HiFi reads. Note that mismatched bases are not shown in reads at this zoom level, but can be seen as coloured vertical lines in the coverage tracks above the read alignments. This region is orthologous to the region shown in Figure 4 and Supplementary Figure 14.

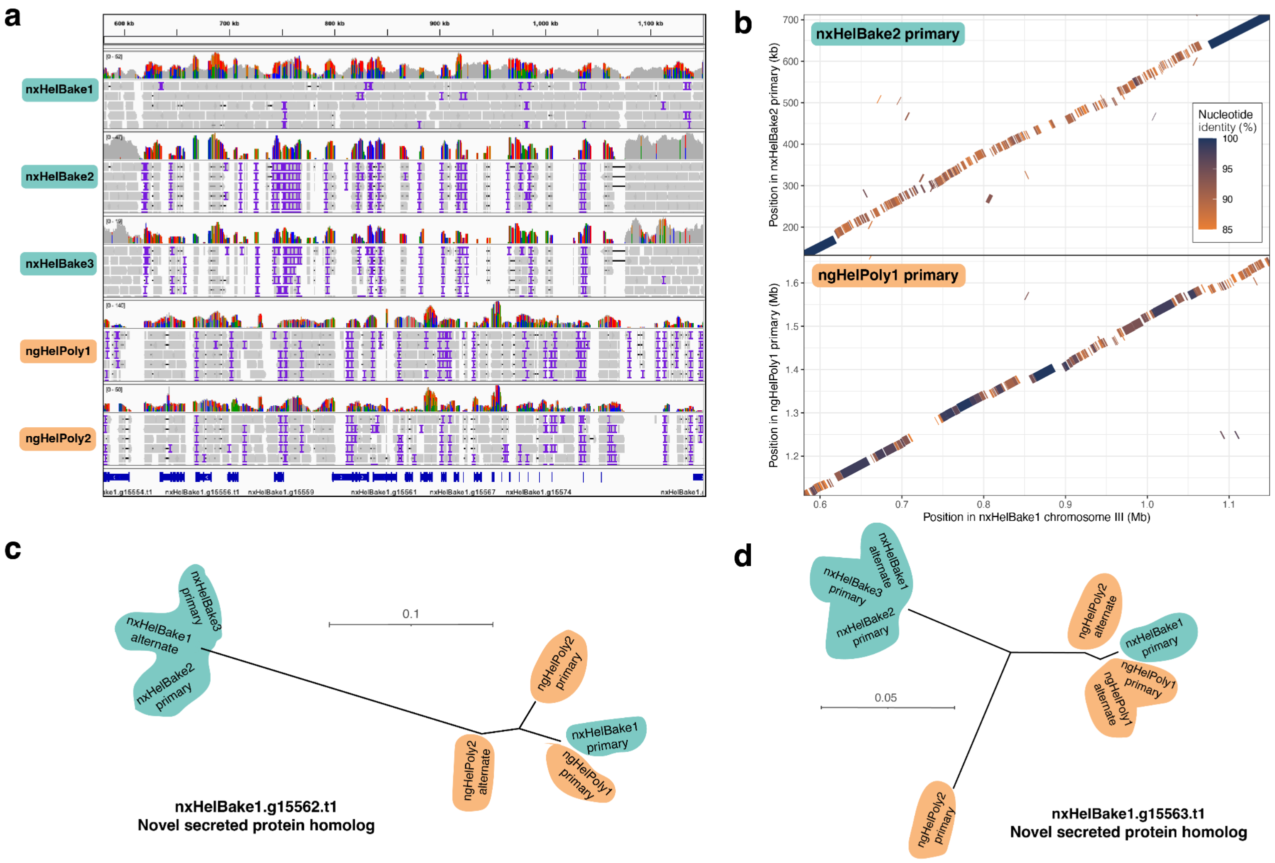


## Supplementary Figure 17: Trans-specific polymorphism in novel secreted proteins homologs

(a) PacBio HiFi read alignments to the nxHelBake1.1 reference genome in a ~570 kb region on chromosome III (0.58-1.15 Mb). The top panel shows the coverage and the bottom panel shows aligned PacBio HiFi reads. The coloured vertical lines indicate mismatched bases at that position. (b) Nucleotide alignments between the alternate *H. bakeri* haplotype (represented by nxHelBake2 primary), ngHelPoly1 primary and the nxHelBake1 reference haplotype. Repetitive alignments are not shown. Gene trees of (c) nxHelBake1.g15562.t1 and (d) nxHelBake1.g15563.t1 showing evidence of haplotype sharing between nxHelBake1 primary and various *H. polygyrus* haplotypes. Trees were inferred using IQ-TREE under the LG+Γ substitution model. Scale is shown in substitutions per site. Outgroup not shown. Source data for figures b-d can be found in the GitHub repository.

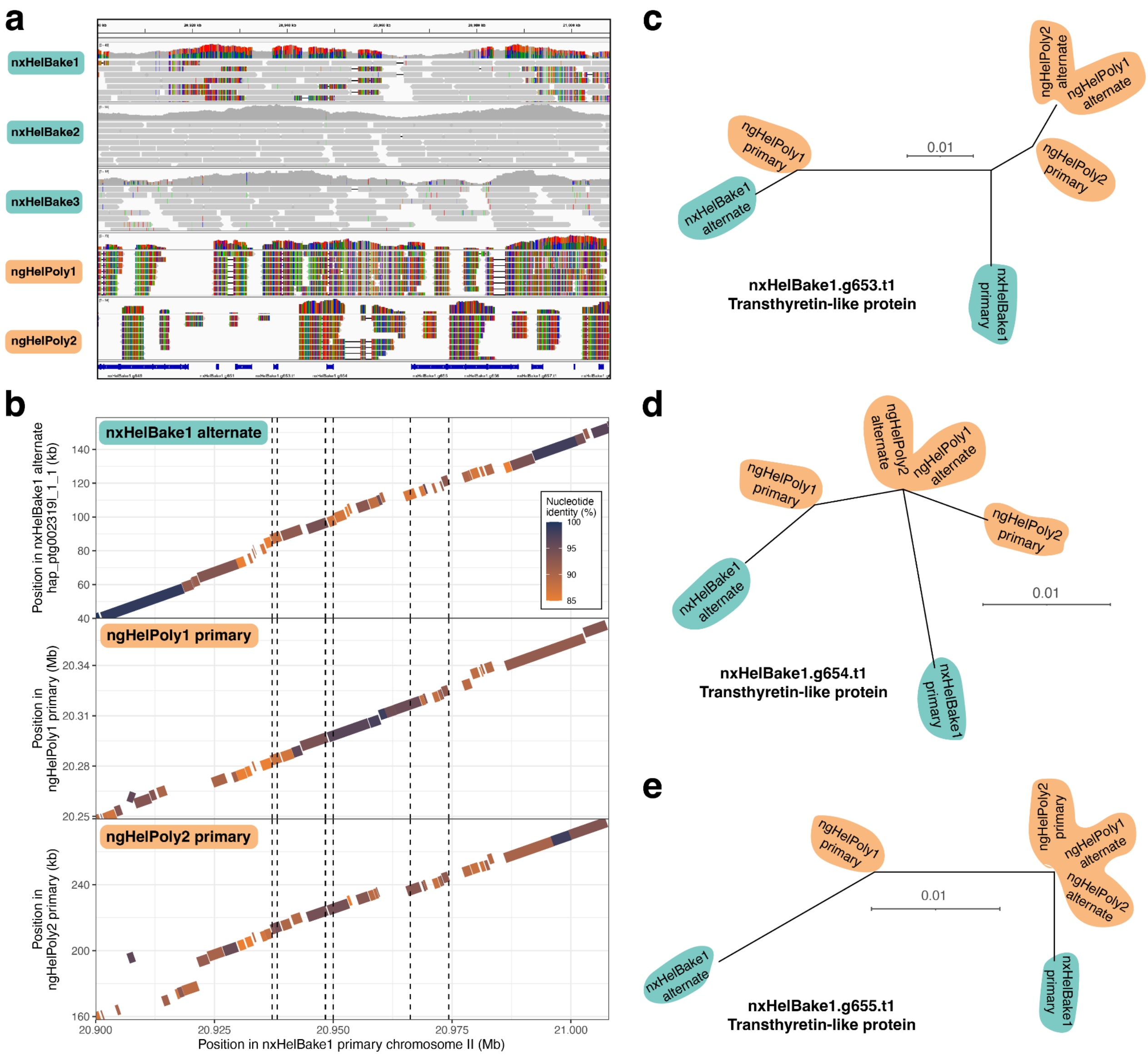


**Supplementary Figure 18: Trans-specific polymorphism in a region containing multiple transthyretin-like proteins**

(a) PacBio HiFi read alignments to the nxHelBake1.1 reference genome in a 177 kb region on chromosome II (20.9 - 21.1 Mb). The top panel shows the coverage and the bottom panel shows aligned PacBio HiFi reads. The coloured vertical lines indicate mismatched bases at that position. (b) Nucleotide alignments between the alternate *H. bakeri* haplotype (represented by nxHelBake1 alternate), ngHelPoly1 primary, ngHelPoly2 primary and the nxHelBake1 reference haplotype. Repetitive alignments are not shown. Gene trees of (c) nxHelBake1.g653.t1, (d) nxHelBake1.g654.t1, and (e) nxHelBake1.g655.t1 showing evidence of haplotype sharing between *H. bakeri* and *H. polygyrus* haplotypes. Tree inferred using IQ-TREE under the LG+Γ substitution model. Scale is shown in substitutions per site. Outgroup not shown. Source data for figures b-e can be found in the GitHub repository.

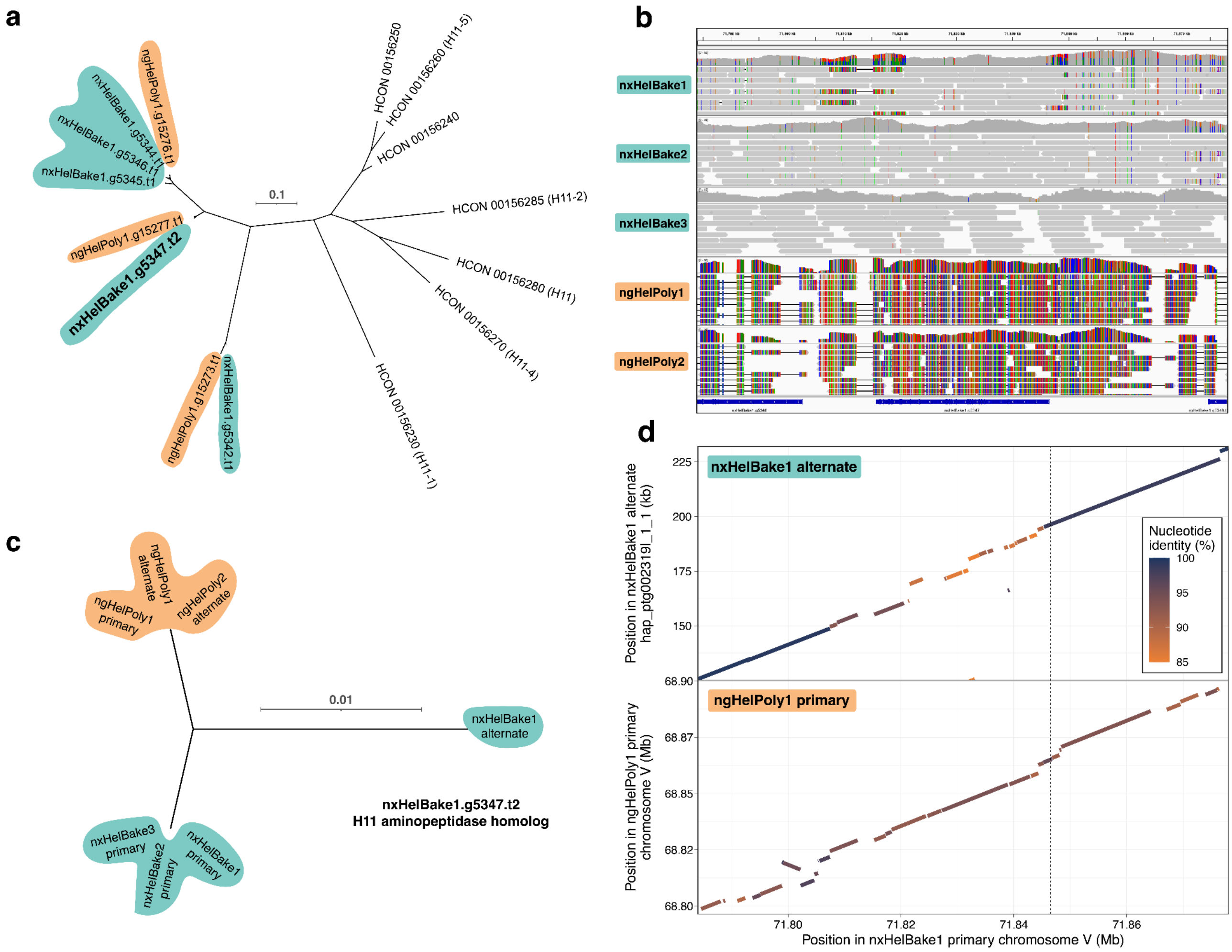


**Supplementary Figure 19: Trans-specific polymorphism in a homolog of H11 aminopeptidase**

(a) Gene tree of H11 homologs in *H. bakeri*, *H. polygyrus* and *H. contortus*. Tree inferred using IQ-TREE under the LG+Γ substitution model. Scale is shown in substitutions per site. nxHelBake1.g5347.t2 is highlighted in bold. The names of *H. contortus* homologs are shown in parentheses which were inferred using phylogenetic relationships to previously published H11 protein sequences downloaded from NCBI. (b) PacBio HiFi read alignments to the nxHelBake1.1 reference genome in a 94 kb region on chromosome V (71.78-71.88 Mb). The top panel shows the coverage and the bottom panel shows aligned PacBio HiFi reads. The coloured vertical lines indicate mismatched bases at that position. (c) Gene tree of nxHelBake1.g5347.t2 and its homologs in the other *H. bakeri* and *H. polygyrus* haplotypes. Tree inferred using IQ-TREE under the LG+Γ substitution model. Scale is shown in substitutions per site. Outgroup not shown. (d) Nucleotide alignments between the alternate *H. bakeri* haplotype (represented by nxHelBake1 alternate), ngHelPoly1 primary and the nxHelBake1 reference haplotype. Repetitive alignments are not shown. Source data for figures a, c, and d can be found in the GitHub repository.

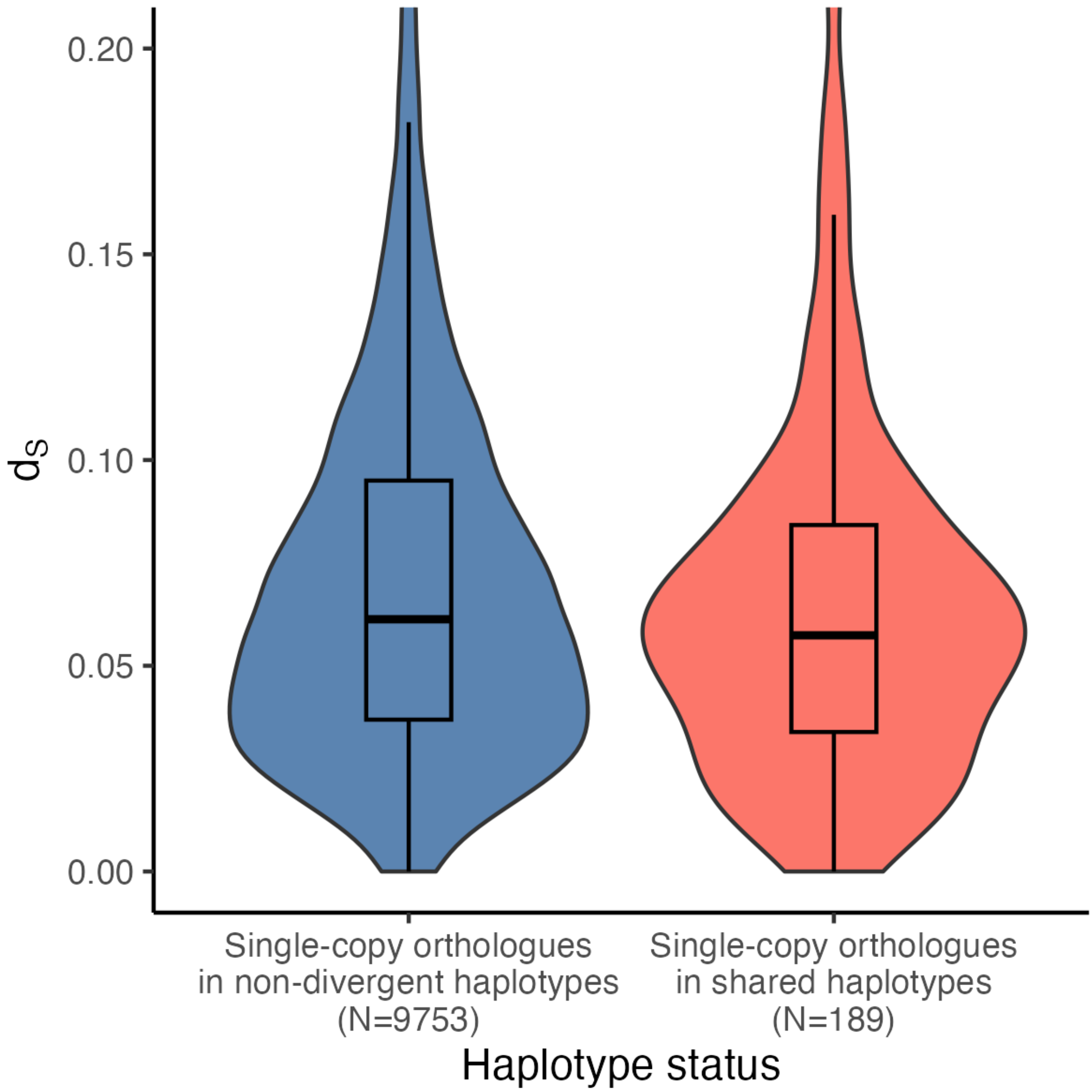


**Supplementary Figure 20: Shared haplotypes show similar levels of divergence to the genome-wide average**

The average synonymous site divergence ($d_S$) for one-to-one orthologs in shared haplotypes (shared; $N$=189) and for all other orthologues (non-shared; $N$=9,753). A two-sided Wilcoxon test suggests no statistical difference between the mean $d_S$ in shared and non-shared haplotypes ($p$-value = 0.1362). Source data for this figure can be found in the Source Data file.

## Supplementary Table 1: Single-worm sequencing and assembly metrics

| Individual ToLID | nxHelBake1 | nxHelBake2 | nxHelBake3 | ngHelPoly1[1] | ngHelPoly2 |
|---|---|---|---|---|---|
| **Species** | *H. bakeri* | *H. bakeri* | *H. bakeri* | *H. polygyrus* | *H. polygyrus* |
| **Sex[2]** | Male | Male | Male | Female | Male |
| **Read count (n)** | 1,884,536 | 2,422,012 | 626,996 | 3,502,508 | 2,768,938 |
| **Bases (Gb)** | 20.7 | 24.1 | 6.2 | 33.9 | 28.1 |
| **Read N50 (kb)** | 11.1 | 9.9 | 10.9 | 9.6 | 10.1 |
| **Coverage (x)[3]** | 31.8 | 37.1 | 9.5 | 52.2 | 43.2 |
| **% PCR duplicates** | 15.6 | 18.3 | 5.9 | 12.6 | 14 |
| **Unique read count** | 1,590,050 | 1,979,754 | 589,768 | 3,061,678 | 2,380,870 |
| **Unique bases (Gb)** | 17.3 | 19.6 | 5.7 | 29.4 | 24 |
| **Unique read N50 (kb)** | 11 | 9.8 | 9.8 | 9.6 | 10.1 |
| **Unique coverage (x)[3]** | 26.7 | 30.2 | 8.8 | 45.3 | 36.9 |
| **Primary assembly span (Mb)** | 654.6 | 656.5 | 572.8 | 658.3 | 620 |
| **Contigs in primary assembly (n)** | 3,585 | 4,235 | 8,129 | 7,427 | 7,366 |
| **Primary assembly N50 (kb)** | 314 | 266.8 | 102.3 | 310.2 | 136.7 |
| **BUSCO completeness of primary assembly (%)** | 91.5 | 91.3 | 80.5 | 92.9 | 87.7 |
| **BUSCO duplication of primary assembly (%)** | 1.7 | 1.6 | 1.7 | 3 | 3 |
| **Alternate assembly span (Mb)** | 304.5 | 328.3 | 154 | 623.8 | 464.7 |
| **Contigs in alternate assembly (n)** | 9,763 | 10,900 | 6,714 | 14,214 | 12,604 |
| **Alternate assembly N50 (kb)** | 37.1 | 38.9 | 24.8 | 71.8 | 50.7 |
| **BUSCO completeness of alternate assembly (%)** | 38.3 | 40 | 17.7 | 83.2 | 65.7 |
| **BUSCO duplication of alternate assembly (%)** | 1.3 | 1.6 | 0.3 | 3.5 | 2.3 |

1. The library for this individual was run on two PacBio Sequel IIe flow cells; the metrics reported represent data from both flow cells combined.

2. Sex was inferred based on coverage of the X chromosome (males are hemizygous for X and therefore have half coverage).

3. Based on an estimated genome size of 650 Mb.

## Supplementary Table 2: Protein-coding gene prediction metrics

| *H. bakeri nxHelBake1.1* | | | | |
|---|---|---|---|---|
| | **BRAKER1 (RNA-seq)** | **BRAKER2 (homology)** | **TSEBRA** | **TSEBRA+PASAx2** |
| Number of genes | 17843 | 14512 | 19299 | 19117 |
| Number of transcripts | 20425 | 15432 | 22188 | 28195 |
| Number of single exon genes | 1373 | 2265 | 2438 | 2406 |
| Percent single exon genes | 7.69% | 15.61% | 12.63% | 12.59% |
| Number of transcripts with 5' UTRs | 0 | 0 | 0 | 11008 |
| Number of transcripts with 3' UTRs | 0 | 0 | 0 | 14811 |
| BUSCO completeness (%) | 92.50% | 89.60% | 92.70% | 92.80% |
| Number of complete BUSCOs | 2895 | 2805 | 2903 | 2905 |
| Number of fragmented BUSCOs | 42 | 85 | 46 | 47 |
| Number of missing BUSCOs | 194 | 241 | 182 | 179 |
| Number of single-copy orthologues with H. contortus | 8619 | 7461 | 8725 | 8725 |
| Number of single-copy orthologues that are within 10% of the H. contortus length | 6129 | 3526 | 6303 | 6427 |
| Number of genes in orthogroups with H. contortus | 15297 | 11918 | 16200 | 16110 |
| Percent of genes in orthogroups with H. contortus (%) | 85.70% | 82.10% | 83.90% | 84.30% |

| *H. polygyrus ngHelPoly1.1* | | | | |
|---|---|---|---|---|
| | **BRAKER1 (RNA-seq)** | **BRAKER2 (homology)** | **TSEBRA** | **TSEBRA+PASAx2** |
| Number of genes | 19576 | 15370 | 20735 | 20622 |
| Number of transcripts | 21754 | 16369 | 23651 | 24144 |
| Number of single exon genes | 2482 | 2661 | 3322 | 3277 |
| Percent single exon genes | 12.68% | 17.31% | 16.02% | 15.89% |
| Number of transcripts with 5' UTRs | 0 | 0 | 0 | 2843 |
| Number of transcripts with 3' UTRs | 0 | 0 | 0 | 7215 |
| BUSCO completeness (%) | 94.20% | 91.20% | 93.90% | 94.00% |
| Number of complete BUSCOs | 2948 | 2857 | 2941 | 2944 |
| Number of fragmented BUSCOs | 31 | 65 | 35 | 34 |
| Number of missing BUSCOs | 152 | 209 | 155 | 153 |
| Number of single-copy orthologues with H. contortus | 8496 | 7376 | 8563 | 8578 |
| Number of single-copy orthologues that are within 10% of the H. contortus length | 6210 | 3508 | 6260 | 6362 |
| Number of genes in orthogroups with H. contortus | 16853 | 12463 | 17376 | 17322 |
| Percent of genes in orthogroups with H. contortus (%) | 86.10% | 81.10% | 83.80% | 84.00% |

## Supplementary Table 3: Heterozygosity in the *H. bakeri* and *H. polygyrus* genomes

| | | nxHelBake1 | nxHelBake2 | nxHelBake3 | ngHelPoly1 | ngHelPoly2 |
|---|---|---|---|---|---|---|
| **Whole genome** | **Non-repeat span (Mb)** | 229.13 | 229.13 | 229.13 | 236.51 | 236.51 |
| | **Non-repeat SNPs** | 367,123 | 506,973 | 310,450 | 1,521,094 | 1,632,007 |
| | **Non-repeat SNP density** | 0.0016 | 0.0022 | 0.0014 | 0.0064 | 0.0069 |
| | **Average per bp** | 624.12 | 451.96 | 738.06 | 155.49 | 144.92 |
| | **Non-repeat homozygous span** | 166.21 | 154.36 | 167.88 | 38.15 | 56.58 |
| | **Non-repeat homozygous proportion** | 72.54% | 67.37% | 73.27% | 16.13% | 23.92% |
| **Autosomes only** | **Non-repeat span (Mb)** | 197.71 | 197.71 | 197.71 | 204.19 | 204.19 |
| | **Non-repeat SNPs (n)** | 363,853 | 502,869 | 307,446 | 1,426,617 | 1,624,748 |
| | **Non-repeat SNP density** | 0.0018 | 0.0025 | 0.0016 | 0.007 | 0.008 |
| | **Average per bp** | 543.37 | 393.16 | 643.06 | 143.13 | 125.68 |
| | **Non-repeat homozygous span** | 138.42 | 126.8 | 138.85 | 31.5 | 30.54 |
| | **Non-repeat homozygous proportion** | 70.01% | 64.14% | 70.23% | 15.42% | 14.96% |

**Supplementary Table 4: *H. bakeri* hyper-divergent haplotype metrics**

| Haplotype assembly | Number of hyper-divergent haplotypes | Span of hyper-divergent haplotypes (Mb) | Proportion of genome (%) | Number of hyper-divergent genes[1] | Proportion of gene set (%) |
|---|---|---|---|---|---|
| nxHelBake1 alternate | 683 | 23.4 | 3.60% | 665 | 3.50% |
| nxHelBake2 alternate | 468 | 17.1 | 2.60% | 542 | 2.80% |
| nxHelBake2 primary | 861 | 27.4 | 4.20% | 706 | 3.70% |
| nxHelBake3 alternate | 153 | 4.5 | 0.70% | 132 | 0.70% |
| nxHelBake3 primary | 720 | 22.2 | 3.40% | 619 | 3.20% |
| **Total[2]** | **1703** | **62** | **9.60%** | **1734** | **9.10%** |

1. Hyper-divergent genes are those where ≥ 50% of their length was covered by a hyper-divergent haplotype

2. Total values are the result of merging overlapping hyper-divergent haplotypes from all

**Supplementary Table 5: GO terms significantly enriched in hyper-divergent haplotypes**

| GO ID | Description | Count in genome | Count in HD haplotypes | Expected | p-value (weight01) | p-value (classic Fisher)[1] |
|---|---|---|---|---|---|---|
| *Molecular function* | | | | | | |
| GO:0005254 | chloride channel activity | 27 | 7 | 2.24 | 0.0075 | 0.0054 |
| GO:0008191 | metalloendopeptidase inhibitor activity | 6 | 3 | 0.5 | 0.0094 | 0.0094 |
| GO:0051082 | unfolded protein binding | 24 | 6 | 1.99 | 0.0118 | 0.0118 |
| GO:0032217 | riboflavin transmembrane transporter activity | 3 | 2 | 0.25 | 0.0195 | 0.0195 |
| GO:0009922 | fatty acid elongase activity | 9 | 3 | 0.75 | 0.0328 | 0.0328 |
| GO:0030414 | peptidase inhibitor activity | 87 | 14 | 7.23 | 0.0363 | 0.0119 |
| *Biological process* | | | | | | |
| GO:1990456 | mitochondrion-endoplasmic reticulum membrane tethering | 2 | 2 | 0.17 | 0.0076 | 0.0076 |
| GO:0006801 | superoxide metabolic process | 6 | 3 | 0.52 | 0.0108 | 0.0108 |
| GO:0032012 | regulation of ARF protein signal transduction | 7 | 3 | 0.61 | 0.0177 | 0.0177 |
| GO:0032218 | riboflavin transport | 3 | 2 | 0.26 | 0.0215 | 0.0215 |
| GO:0007606 | sensory perception of chemical stimulus | 41 | 8 | 3.58 | 0.0232 | 0.0232 |
| GO:0060828 | regulation of canonical Wnt signaling pathway | 9 | 3 | 0.79 | 0.0373 | 0.0373 |
| GO:0051560 | mitochondrial calcium ion homeostasis | 4 | 2 | 0.35 | 0.0406 | 0.0406 |
| *Cellular component* | | | | | | |
| GO:0009986 | cell surface | 61 | 15 | 5.02 | 7.60E-05 | 7.60E-05 |
| GO:0044233 | mitochondria-associated endoplasmic reticulum membrane | 2 | 2 | 0.16 | 0.0067 | 0.0067 |
| GO:0000159 | protein phosphatase type 2A complex | 3 | 2 | 0.25 | 0.0191 | 0.0191 |
| GO:0000814 | ESCRT II complex | 3 | 2 | 0.25 | 0.0191 | 0.0191 |
| GO:0016589 | NURF complex | 3 | 2 | 0.25 | 0.0191 | 0.0191 |
| GO:0005744 | TIM23 mitochondrial import inner membrane translocase complex | 4 | 2 | 0.33 | 0.0362 | 0.0362 |
| GO:0016021 | integral component of membrane | 854 | 83 | 70.26 | 0.0419 | 0.0324 |

1. Fisher's exact test used in TopGo is a one-sided test.

**Supplementary Table 6: Accessions of the data used in gene prediction and phylogenomic analyses**

| Species | Category | Source | BioProject |
|---|---|---|---|
| *Ancylostoma caninum* | Strongylomorpha | WBPS (version 17) | PRJNA72585 |
| *Ancylostoma ceylanicum* | Strongylomorpha | WBPS (version 17) | PRJNA231479 |
| *Ancylostoma duodenale* | Strongylomorpha | WBPS (version 17) | PRJNA72581 |
| *Angiostrongylus cantonensis* | Strongylomorpha | WBPS (version 17) | PRJNA350391 |
| *Angiostrongylus costaricensis* | Strongylomorpha | WBPS (version 17) | PRJEB494 |
| *Angiostrongylus vasorum* | Strongylomorpha | WBPS (version 17) | PRJNA663250 |
| *Caenorhabditis elegans* | Outgroup | WBPS (version 17) | PRJNA13758 |
| *Cylicostephanus goldi* | Strongylomorpha | WBPS (version 17) | PRJEB498 |
| *Dictyocaulus viviparus* | Strongylomorpha | WBPS (version 17) | PRJNA72587 |
| *Haemonchus contortus* | Strongylomorpha | WBPS (version 17) | PRJEB506 |
| *Haemonchus placei* | Strongylomorpha | WBPS (version 17) | PRJEB509 |
| *Heligmosomoides bakeri* | Strongylomorpha | this work | PRJEB57615 |
| *Heligmosomoides polygyrus* | Strongylomorpha | this work | PRJEB57641 |
| *Heterorhabditis bacteriophora* | Outgroup | WBPS (version 17) | PRJNA13977 |
| *Heterorhabditis indica* | Outgroup | NCBI | PRJNA720543 |
| *Necator americanus* | Strongylomorpha | WBPS (version 17) | PRJNA72135 |
| *Nippostrongylus brasiliensis* | Strongylomorpha | WBPS (version 17) | PRJEB511 |
| *Oesophagostomum dentatum* | Strongylomorpha | WBPS (version 17) | PRJNA72579 |
| *Parelaphostrongylus tenuis* | Strongylomorpha | NCBI | PRJNA729714 |
| *Strongylus vulgaris* | Strongylomorpha | WBPS (version 17) | PRJEB531 |
| *Teladorsagia circumcincta* | Strongylomorpha | WBPS (version 17) | PRJNA72569 |

## Supplementary Table 7: *Heligmosomum* genome assembly metrics

| **Individual** | **Hm2** | **Hm16** |
|---|---|---|
| Reads in read set (n) | 250,957,362 | 231,078,046 |
| Bases in read set (Gb) | 31.4 | 28.9 |
| Estimated coverage (x) | 54.5 | 50.2 |
| Number of merged reads (%) | 83.80% | 79.30% |
| Assembly span (Mb) | 641 | 645.9 |
| Scaffolds in assembly (n) | 763,947 | 714,693 |
| Scaffold N50 (kb) | 2,577 | 3,051 |
| BUSCO completeness (%) | 42 | 42 |
| BUSCO duplication (%) | 0.7 | 0.7 |

## Supplementary Table 8: Genome assembly accession numbers

| Individual | Assembly | BioProject |
|---|---|---|
| nxHelBake1 | Primary | PRJEB57615 |
| nxHelBake1 | Alternate | PRJEB57616 |
| nxHelBake2 | Primary | PRJEB67323 |
| nxHelBake2 | Alternate | PRJEB67322 |
| nxHelBake3 | Primary | PRJEB67321 |
| nxHelBake3 | Alternate | PRJEB67320 |
| ngHelPoly1 | Primary | PRJEB57641 |
| ngHelPoly1 | Alternate | PRJEB57642 |
| ngHelPoly2 | Primary | PRJEB67327 |
| ngHelPoly2 | Alternate | PRJEB67326 |
