# Supplementary material for: Ancient diversity in host-parasite interaction genes in a model parasitic nematode
Source: Nat Commun. 2023 Nov 27;14:7776. doi: 10.1038/s41467-023-43556-w (PMC10682056; doi:10.1038/s41467-023-43556-w)
Supplement: Supplementary file 3 — Description of Additional Supplementary Files [file 41467_2023_43556_MOESM3_ESM.pdf]

Description of Additional Supplementary Files

Filename: Supplementary Data 1
Description: Excel spreadsheet containing transcript identifiers corresponding to genes in the *H. bakeri* nxHelBake1.1 genome that show evidence of haplotype sharing along with their Pfam domains (separated by a semi-colon if multiple domains).
