# Supplementary material for: Ancient diversity in host-parasite interaction genes in a model parasitic nematode
Source: Nat Commun. 2023 Nov 27;14:7776. doi: 10.1038/s41467-023-43556-w (PMC10682056; doi:10.1038/s41467-023-43556-w)
Supplement: Supplementary file 5 — Reporting Summary [file 41467_2023_43556_MOESM5_ESM.pdf]

nature portfolio

Corresponding author(s): Lewis Stevens

Last updated by author(s): Oct 3, 2023

Reporting Summary

Nature Portfolio wishes to improve the reproducibility of the work that we publish. This form provides structure for consistency and transparency in reporting. For further information on Nature Portfolio policies, see our Editorial Policies and the Editorial Policy Checklist.

Statistics

For all statistical analyses, confirm that the following items are present in the figure legend, table legend, main text, or Methods section.

n/a	Confirmed	
☐	☒	The exact sample size (n) for each experimental group/condition, given as a discrete number and unit of measurement
☒	☐	A statement on whether measurements were taken from distinct samples or whether the same sample was measured repeatedly
☐	☒	The statistical test(s) used AND whether they are one- or two-sided<br>*Only common tests should be described solely by name; describe more complex techniques in the Methods section.*
☒	☐	A description of all covariates tested
☒	☐	A description of any assumptions or corrections, such as tests of normality and adjustment for multiple comparisons
☒	☐	A full description of the statistical parameters including central tendency (e.g. means) or other basic estimates (e.g. regression coefficient) AND variation (e.g. standard deviation) or associated estimates of uncertainty (e.g. confidence intervals)
☐	☒	For null hypothesis testing, the test statistic (e.g. F, t, r) with confidence intervals, effect sizes, degrees of freedom and P value noted<br>*Give P values as exact values whenever suitable.*
☒	☐	For Bayesian analysis, information on the choice of priors and Markov chain Monte Carlo settings
☒	☐	For hierarchical and complex designs, identification of the appropriate level for tests and full reporting of outcomes
☒	☐	Estimates of effect sizes (e.g. Cohen's d, Pearson's r), indicating how they were calculated

Our web collection on statistics for biologists contains articles on many of the points above.

Software and code

Policy information about availability of computer code

Data collection	We did not used software during data collection.
Data analysis	All third-party software used in the manuscript is described in the methods alongside relevant citations and version numbers and list below.<br>All custom code used is available in the public GitHub repository (https://github.com/lstevens17/heligmosomoides_MS). The repository includes descriptions of what the code does and how it was used.<br>Third-party software and versions (where available):<br>lima v2.6.0<br>pbmarkdup v1.0.2<br>HiFiAdapterFilt<br>Jellyfish 2.3.0<br>GenomeScope 2.0<br>hifiasm v0.16.1-r375<br>bwa mem 0.7.17-r1188<br>piccard 2.27.1-0<br>YaHS 1.1a<br>BlobToolKit 2.6.5<br>minimap2 2.24-r1122<br>MitoHiFi 2.2<br>purge_dups 1.2.5<br>Seqkit v2.1.0<br>BUSCO 5.2.2

Merqury 1.3
PretextView 0.2.5
Juicer 2.0
Juicebox 1.11.08
BEDtools v2.30.0
mosdepth 0.3.3
fasta_windows 0.2.4.
IsoSeq refine 3.8.2
IsoSeq cluster 3.8.2
earlGrey v1.2
MAFFT v7.487
trimAl v1.4. Rev15
AliView v1.28
EMBOSS cons v6.6.0.0
CIAlign 1.1.0
TE-Aid
cd-hit-est v.4.8.1
RepeatModeler2 v2.0.3
RepeatMasker 4.1.2-p1
BRAKER1 2.1.6
STAR 2.7.10a
SAMtools 1.3
TSEBRA v1.0.3
PASA 2.5.2
AGAT 0.8.1
OrthoFinder 2.5.4
fastp 0.23.2
VSEARCH v2.22.1
SPAdes 3.12.0
FSA 1.15.9
IQ-TREE v2.2.0.3
Astral v5.7.8
nhmmer v3.3.2
pal2nal v14
PAML 4.9j
BCFtools 1.15
VCFtools 0.1.16
pixy 1.2.7.beta1
DeepVariant 1.4.0
BCFtools 1.15
NUCmer 3.1
paftools 2.24-r1122
InterProScan 5.54-87.0
TopGO R package 2.50.0
miniprot 0.7-r20

For manuscripts utilizing custom algorithms or software that are central to the research but not yet described in published literature, software must be made available to editors and reviewers. We strongly encourage code deposition in a community repository (e.g. GitHub). See the Nature Portfolio guidelines for submitting code & software for further information.

Data

Policy information about availability of data

All manuscripts must include a data availability statement. This statement should provide the following information, where applicable:

- Accession codes, unique identifiers, or web links for publicly available datasets
- A description of any restrictions on data availability
- For clinical datasets or third party data, please ensure that the statement adheres to our policy

The H. bakeri nxHelBake1.1 and H. polygyrus ngHelPoly1.1 reference genomes have been deposited in ENA under the BioProject accessions PRJEB57615 [https://www.ebi.ac.uk/ena/browser/view/PRJEB57615] and PRJEB57641 [https://www.ebi.ac.uk/ena/browser/view/PRJEB57641], respectively. Accession numbers for the other assemblies generated as part of this study (alternate assemblies and assemblies for the other three individuals) are available in Supplementary Table 8. PacBio and HiC data have been deposited in ENA under the BioProject accessions PRJEB46574 [https://www.ebi.ac.uk/ena/browser/view/PRJEB46574] and PRJEB36817 [https://www.ebi.ac.uk/ena/browser/view/PRJEB36817]. The H. mixtum raw data and genome assembly have been deposited in ENA under the BioProject PRJEB61185 [https://www.ebi.ac.uk/ena/browser/view/PRJEB61185]. The H. bakeri RNA-seq reads used in gene prediction are available in ENA under the BioProject PRJNA486010 [https://www.ebi.ac.uk/ena/browser/view/PRJNA486010]. The H. polygyrus short-read RNA-seq reads have been deposited in ENA under the BioProject PRJEB61184 [https://www.ebi.ac.uk/ena/browser/view/PRJEB61184]. Accession numbers for the genome assemblies and annotations used for gene predictions and phylogenomic analysis are available in Supplementary Table 6. Large data files associated with this manuscript, including VCFs, gene annotation files, curated repeat libraries, and Newick files have been deposited in Zenodo under the DOI 10.5281/zenodo.8403377 [https://zenodo.org/doi/10.5281/zenodo.8403377]. Data files associated with the manuscript can be found in the GitHub (https://github.com/lstevens17/heligmosomoides_MS), which has been accessioned in Zenodo under the DOI 10.5281/zenodo.10092962 [https://doi.org/10.5281/zenodo.10092962]. Source data associated with the figures and tables can be found in the Source Data file, the GitHub repository, and in ENA.

Research involving human participants, their data, or biological material

Policy information about studies with human participants or human data. See also policy information about sex, gender (identity/presentation), and sexual orientation and race, ethnicity and racism.

Reporting on sex and gender	Our study did not involve human participants or human data
Reporting on race, ethnicity, or other socially relevant groupings	Our study did not involve human participants or human data
Population characteristics	Our study did not involve human participants or human data
Recruitment	Our study did not involve human participants or human data
Ethics oversight	Our study did not involve human participants or human data

Note that full information on the approval of the study protocol must also be provided in the manuscript.

Field-specific reporting

Please select the one below that is the best fit for your research. If you are not sure, read the appropriate sections before making your selection.

☒ Life sciences ☐ Behavioural & social sciences ☐ Ecological, evolutionary & environmental sciences

For a reference copy of the document with all sections, see nature.com/documents/nr-reporting-summary-flat.pdf

Life sciences study design

All studies must disclose on these points even when the disclosure is negative.

Sample size	In our GO term enrichment analysis that compared divergence between shared haplotypes and non-divergent regions of the genome, the sample sizes were determined by the number of genes that belonged to each group. We therefore did not perform sample size calculations and we used the maximum number of genes available to us. Samples sizes are stated in all case.
Data exclusions	No data were excluded from the analysis.
Replication	Replicating our study (by resequencing individuals and reassembling and reanalysing the genomes) would be cost-prohibitive.
Randomization	Randomization was not relevant to this study because we did not carry out a randomised control trial.
Blinding	Blinding was not relevant to this study because we did not carry out a randomised control trial.

Reporting for specific materials, systems and methods

We require information from authors about some types of materials, experimental systems and methods used in many studies. Here, indicate whether each material, system or method listed is relevant to your study. If you are not sure if a list item applies to your research, read the appropriate section before selecting a response.

Materials & experimental systems

n/a	Involved in the study	
☒	☐	Antibodies
☒	☐	Eukaryotic cell lines
☒	☐	Palaeontology and archaeology
☐	☒	Animals and other organisms
☒	☐	Clinical data
☒	☐	Dual use research of concern
☒	☐	Plants

Methods

n/a	Involved in the study	
☒	☐	ChIP-seq
☒	☐	Flow cytometry
☒	☐	MRI-based neuroimaging

Animals and other research organisms

Policy information about studies involving animals; ARRIVE guidelines recommended for reporting animal research, and Sex and Gender in Research

Laboratory animals	CBA × C57BL/6 F1 (CBF1) mice were used. The strain
Wild animals	A. sylvaticus were trapped on ten grids over two sites in a three-weekly rotation from May to December 2019. The two sites were based within 14 km from Edinburgh, Scotland: one in Penicuik Estate (55 48'56.5"N 3 15'23.1"W) and the other in Hewan Wood, near Loanhead (55 52'09.3"N 3 08'34.4"W). Trapping occurred three days a week. Each site contained five 60 m x 60 m grids. Traps were baited with birdseed, carrot and cotton bedding. These were set at dusk each day and checked for animals the following morning. Each animal was weighed, sexed, measured, given a fat score (1 to 5), and checked for ectoparasites. All traps were kept to harvest the faecal matter. A subset of the trapped A. sylvaticus were sacrificed using cervical dislocation and confirmed by exsanguination. The gastrointestinal tracts were removed and the small intestine was examined for adult H. polygyrus, which were carefully removed with forceps, washed, and maintained in serum-free medium in vitro as described previously (Johnston et al. 2015). Individual H. polygyrus were stored in cryovials at -80°C until needed. Individuals collected in Hewan Wood were used for single nematode sequencing (see below). Six co-housed laboratory-reared wild wood mice (A. sylvaticus) aged 6 to 8 weeks, were administered 150 L3 per 150 µl H20 via oral gavage on day 0. These animals were checked daily and had access to food and water ad libitum. From day 9 to day 21 post-infection, the bedding of the mice was collected daily and fresh bedding was supplied. The soiled bedding was sieved to collect all the faecal pellets and these pellets were mixed with inactivated charcoal as detailed above. On day 21 post-infection, the mice were culled via cervical dislocation and confirmation was by exsanguination. The GI tracts were dissected and any adult nematodes found were processed as above.<br><br>Bank voles were live-trapped in wooden traps with grain and carrot or apple as bait. Each animal was weighed, sexed, measured, and sacrificed by isoflurane overdose and cervical dislocation. We carefully examined intestines under a stereoscopic microscope in search of nematodes of any species.
Reporting on sex	Sex of hosts is not relevant to this study.
Field-collected samples	Six co-housed laboratory-reared wild wood mice (A. sylvaticus) aged 6 to 8 weeks, were administered 150 L3 per 150 µl H20 via oral gavage on day 0. These animals were checked daily and had access to food and water ad libitum. From day 9 to day 21 post-infection, the bedding of the mice was collected daily and fresh bedding was supplied. The soiled bedding was sieved to collect all the faecal pellets and these pellets were mixed with inactivated charcoal as detailed above. On day 21 post-infection, the mice were culled via cervical dislocation and confirmation was by exsanguination. The GI tracts were dissected and any adult nematodes found were processed as above.
Ethics oversight	The University of Edinburgh’s Animal Welfare and Ethical Review Board approved the procedures used for Mus musculus and Apodemus sylvaticus . Local Ethical Committee no. 1 in Warsaw, Poland (decision 304/2012) approved procedures for Myodes glareolus work.

Note that full information on the approval of the study protocol must also be provided in the manuscript.
